# Efficacy and safety of Chinese medicine injections in combination with docetaxel and cisplatin for non-small cell lung cancer: a network meta-analysis

**DOI:** 10.3389/fphar.2023.1277284

**Published:** 2023-12-11

**Authors:** Liangtao Wen, Lixiang Xie, Fengying Gong, Shunan Zhang, Tieju Xi

**Affiliations:** ^1^ General Practice, Shenzhen Hospital of Integrated Traditional Chinese and Western Medicine, Shenzhen, Guangdong, China; ^2^ Traditional Chinese Medicine, Nanfang Hospital, Southern Medical University, Guangdong, China

**Keywords:** Chinese herbal injection, docetaxel in combination with cisplatin, non-small cell lung cancer, network meta-analysis, systematic evaluation

## Abstract

**Background:** Non-small cell lung cancer (NSCLC) poses a serious threat to human health. Several clinical studies have reported the benefits of Chinese herbal injections (CHIs) in combination with docetaxel and cisplatin (DP). This multidimensional network meta-analysis aimed to investigate the preferred regimen of CHIs in combination with DP for the treatment of NSCLC.

**Methods:** Multiple databases were searched to identify randomized controlled trials (RCTs) of CHIs for NSCLC from the database inception to 30 April 2023. Studies that met the inclusion criteria and exhibited good methodological quality were included. Data analysis was conducted using Stata 15.0 and R 4.2.1 software. An odds ratio (OR) was used as the effect size, and the surface under the cumulative ranking curve (SCURA) was employed to rank the evaluated treatments.

**Results:** The network meta-analysis included 85 eligible RCTs, encompassing 6,580 patients and 11 CHIs. Astragalus Injection combined with DP was identified as the most effective regimen for improving the response rate (SUCRAs: 90.25%). Brucea Javanica Oil Milk Injection combined with DP proved most effective in ameliorating the quality of life (SUCRAs: 76.89%). Shenfu Injection combined with DP emerged as the most effective for enhancing CD3^+^ and CD4^+^ (SUCRAs: 93.75%, 88.50%). Kanglaite Injection combined with DP exhibited the best efficacy in improving CD8^+^ (SUCRAs: 88.96%). Brucea Javanica Oil Milk Injection combined with DP was the most potent regimen for enhancing CD4^+^/CD8^+^ (SUCRAs: 93.13%).

**Conclusion:** CHIs in combination with DP outperformed DP alone in NSCLC patients. Astragalus Injection plus DP, Brucea Javanica Oil Milk Injection plus DP, Shenfu Injection plus DP, Kanglaite Injection plus DP, and Brucea Javanica Oil Milk Injection plus DP were significantly effective. However, further multicenter and well-designed RCTs are required to validate our findings.

## 1 Background

According to the most recent data from the World Health Organization’s International Agency for Research on Cancer (IARC), there were 19.29 million new cancer cases and 9.96 million cancer deaths in 2020. Among these, lung cancer is the predominant type, accounting for 11.6% of all cases, and it is the leading cause of cancer-related deaths, responsible for 18.4% of the total (Ettinger et al., 2013; Ferlay et al., 2015; Bray et al., 2018). Lung cancer has a poor prognosis, with a 5-year survival rate of only 16.8% (Ettinger et al., 2013; Ferlay et al., 2015; Bray et al., 2018). It can be categorized based on histology into non-small cell lung cancer (NSCLC) and small cell lung cancer (SCLC) (Miller et al., 2018). NSCLC comprises roughly 85% of all lung cancer cases, and unfortunately, approximately 50% of NSCLC patients are diagnosed at an advanced stage (La Fleur et al., 2019). Early-stage NSCLC is typically treated with surgery-based comprehensive therapy, while intermediate- and late-stage NSCLC is commonly managed with a combination of radiotherapy, chemotherapy, targeted therapy, and immunotherapy. Although these treatments can be effective for some patients in the short term, they often come with a highly toxic physiological environment and a risk of adverse events (Pfister et al., 2004).

In China, the combination of Traditional Chinese Medicine (TCM) and chemotherapy has been widely employed in the treatment of cancer (Qi et al., 2010). Studies have reported the benefits of TCM as an adjuvant therapy for cancer, particularly in terms of slowing down disease progression and alleviating chemotherapy-induced complications and adverse events (Hofseth and Wargovich, 2007; Konkimalla and Efferth, 2008). Chinese Herbal Injections (CHIs), a significant component of Traditional Chinese Medicine (TCM), have seen increased utilization in the management of cancer, particularly in cases of non-small cell lung cancer (NSCLC). Commonly practiced combinations of CHIs with chemotherapy include CHIs combined with vinorelbine plus cisplatin (NP), CHIs combined with gemcitabine plus cisplatin (GP), CHIs combined with paclitaxel plus cisplatin (TP), CHIs combined with paclitaxel plus carboplatin (TC), CHIs combined with pemetrexed plus cisplatin (PP), and CHIs combined with docetaxel plus cisplatin (DP). Several clinical studies have demonstrated that the combination of CHIs with the above-mentioned chemotherapeutic regimens has yielded positive outcomes in the treatment of NSCLC. This approach can effectively regulate the body’s immune function, reduce the toxic side effects of chemotherapeutic drugs, enhance treatment efficacy, improve the prognosis of advanced NSCLC patients, enhance their quality of life (QoL), and extend their survival period (Zhihao, 2016; Yanlin et al., 2019; Guofu et al., 2020; Shaoguang, 2021; Shuping and Xiaobin, 2022; Xing et al., 2022).

Several network meta-analyses of CHIs with NP, GP, or TP have been conducted (Ni et al., 2020a; Ni et al., 2020b; Wang et al., 2021). Nevertheless, the optimal regimen for combining CHIs with DP in the treatment of NSCLC remains uncertain, which could pose challenges for clinicians in clinical practice. Direct comparisons among herbal injections are lacking. In contrast, network meta-analysis (NMA) enables the integration of comparisons made in clinical trials and concurrent interventions to assess their relative efficacy (Salanti et al., 2014). Therefore, this study systematically evaluates the effectiveness and safety of different CHIs combined with DP regimens for NSCLC through NMA. The aim is to provide evidence-based guidance for clinicians in selecting the most appropriate treatment strategy.

## 2 Methodology

The meta-analysis was conducted following the guidelines of the Preferred Reporting Items for Systematic Reviews and Meta-Analysis (PRISMA) and their specific requirements for Network Meta-Analysis (NMA). The study protocol was registered in the International Prospective Register of Systematic Reviews under the registration number CRD42023445523.

### 2.1 Search strategy

In this network meta-analysis (NMA), a comprehensive literature search was carried out in electronic databases, including Web of Science, Embase, PubMed, Cochrane Library, CNKI, Wanfang, CQVIP, and CBMDisc. There were no restrictions on publication year, language, or blinding method. The search period extended from the inception of each database up to 30 April 2023. To identify relevant publications, a combination of MeSH terms and free text search terms was applied in the search, with a focus on three main topics: 1) NSCLC, 2) injection, and 3) randomized controlled trials (RCTs). The specific search strategy used is detailed in Attachment 1. Additionally, the reference lists of published systematic reviews were manually examined to supplement the search and ensure its comprehensiveness.

### 2.2 Inclusion and exclusion criteria

The literature meeting the following criteria was included in this study: Study population: The study encompassed patients diagnosed with NSCLC through cytology or pathology, regardless of gender, age, race, region, or nationality. Patients with NSCLC who had other concurrent tumors were excluded. Interventions: The control group received DP chemotherapy alone, while the study group received a type of CHI combined with DP. Type of study: Only randomized controlled trials (RCTs) were considered. Outcome indicators: The study assessed the following outcome measures: a) Response rate: Response rate was evaluated according to the WHO criteria for assessing solid tumor efficacy. It was categorized into four tiers: Complete response (CR): The complete disappearance of the patient’s visible lesion within 1 month after treatment. Partial response (PR): A reduction of ≥50% in the tumor size of a single lesion or >50% reduction in the vertical diameter product of the two largest tumors in a multiloculated lesion. Stable disease (SD): Refers to no significant change in the patient’s disease for at least 4 weeks and <25% increase or <50% decrease in estimated tumor size. Progressive disease (PD): Involves ≥25% increase in the estimated size of a new or original lesion. In this study, the response rate was calculated as (CR + PR)/total number of patients × 100%. b) Quality of Life (QoL): QoL was assessed using the Karnofsky (Kahn) scale. An increase of ≥10 points in the Karnofsky score after the course of treatment compared to before treatment was considered an improvement, a decrease of ≥10 points indicated a decrease, and an increase or decrease of <10 points was classified as a stable condition. In this study, the improvement rate was calculated as the number of patients with improved QoL/total number of patients × 100%. c) Adverse effects: Adverse effects included leukopenia, hemoglobinopenia, and thrombocytopenia. These were classified into five levels according to the Acute and Subacute Toxicity Criteria for chemotherapeutic drugs developed by WHO: 0, I, II, III, and IV. The incidence of adverse effects was calculated as the number of patients with adverse effects/sample size × 100%. d) Immune cell indicators: Immune cell indicators included CD3^+^, CD4^+^, CD8^+^, and CD4^+^/CD8^+^.

The following types of literature were excluded from this study: 1) Animal or cellular experiments, case reports, research proposals, review articles, letters, editorials, and conference summaries; 2) Literature with missing research data or significant errors; 3) Duplicate publications; 4) Literature for which the full text was not available.

### 2.3 Data extraction

The search results were imported into EndNote, and a two-step screening process was employed. Two investigators independently screened the papers by referring to the inclusion and exclusion criteria based on the title and abstract. Subsequently, full texts were reviewed for a second screening. Any disagreements that arose were resolved through discussion or by seeking advice from a third investigator. Two investigators utilized Excel 2016 to independently extract data from the included literature. This information encompassed the first author, year of publication, country of origin, details on randomization and blinding, descriptions of the interventions and control groups, duration of the treatment, characteristics of the study population, and the outcome indicators. These details were organized in a table of baseline characteristics.

### 2.4 Quality assessment

The Cochrane Risk of Bias Assessment Tool (RoB2.0) (Higgins and Green, 2022) was utilized to evaluate the included studies in six domains: bias due to the randomization process, bias due to deviation from the defined interventions, bias due to missing outcome data, bias due to outcome measurement, bias due to selective reporting of outcomes, and other sources of bias. Two investigators conducted independent assessments for each of these six domains in every study, categorizing them as “low risk,” “high risk,” or “possible risk.” In cases where discrepancies arose, they were resolved through discussion or by seeking input from a third investigator. The results of the evaluations were presented in a risk-of-bias plot.

### 2.5 Statistical analysis

In the analysis, the response rate and Quality of Life (QoL) were presented as risk ratios (RR) with 95% confidence intervals (CI). The immune cell indicators (CD3^+^, CD4^+^, CD8^+^, CD4^+^/CD8^+^) were displayed as weighted mean differences (MD) with 95% CI. To account for heterogeneity between trials, a Bayesian hierarchical random-effects model was initially employed for comparing various treatment options for NSCLC (Dias et al., 2013; Mills et al., 2013). All calculations and graphical representations were generated using R 4.2.1 software and Stata 15.1 software. The Markov chain Monte Carlo (MCMC) simulation was carried out based on the likelihood function and prior assumptions using Bayesian inference with R 4.2.1 software. It involved 500,000 iterations and 20,000 annealing settings to explore the posterior distributions of the examined nodes (Dias et al., 2012; Bois, 2013; Hamra et al., 2013). The node splitting method was used to assess local inconsistency in outcomes with closed loops. A network graph was created to illustrate the relationships among the different treatments. Additionally, a comparison-adjusted funnel plot was utilized to assess potential publication bias (Chaimani et al., 2013; Whegang Youdom et al., 2017). Furthermore, surface under the cumulative ranking probability (SUCRA) values were employed to rank the evaluated treatments. SUCRA values range from 0 to 1, where a higher SUCRA value corresponds to a higher ranking for NSCLC compared to other treatments (Rücker and Schwarzer, 2015; Trinquart et al., 2016). A league table was generated to present the comparisons between each pair of interventions within each outcome.

## 3 Results

**FIGURE 1:**
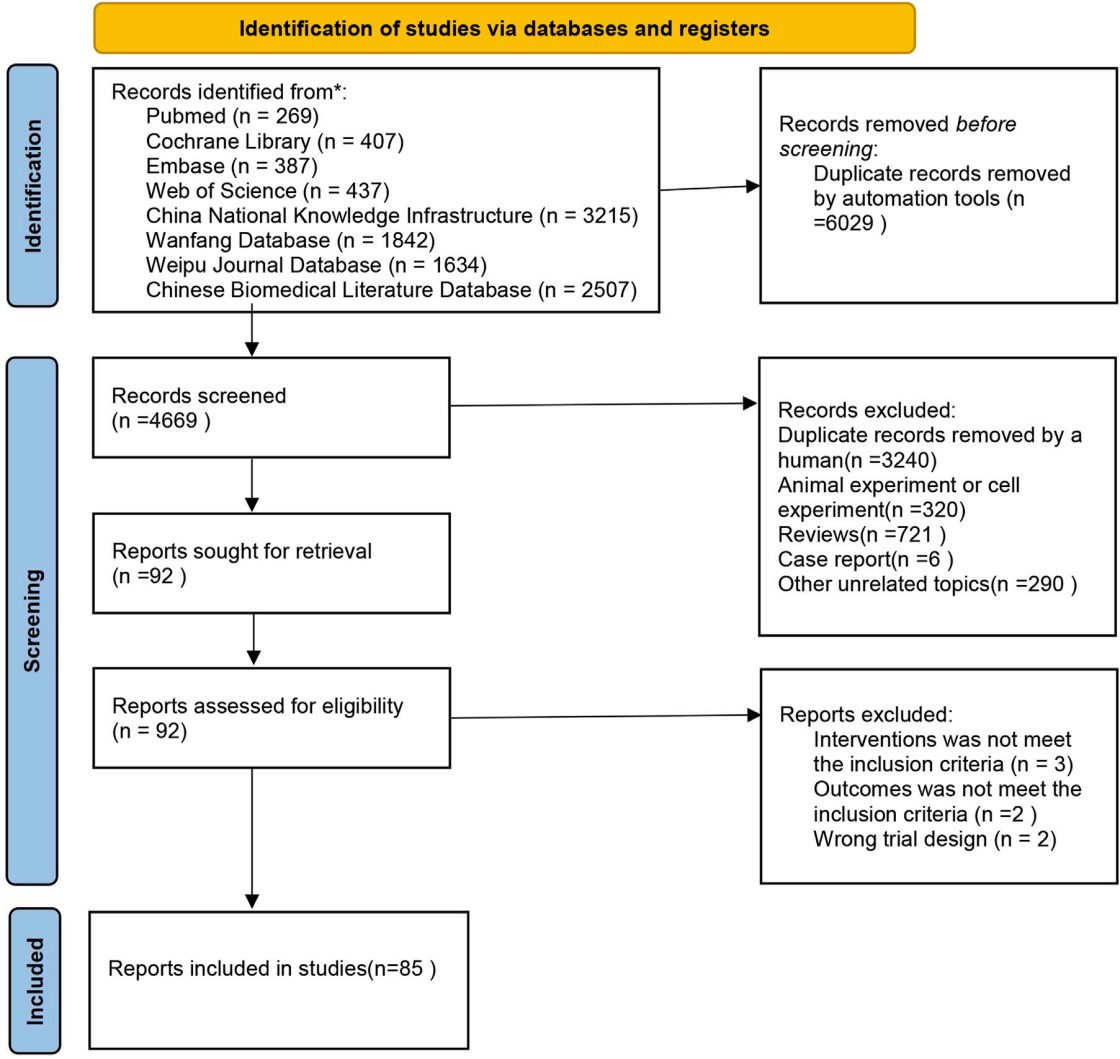
Flowchart.

The initial search produced a total of 10,698 articles. After removing 6,029 duplicates, a further 4,669 articles were excluded following an initial review of titles and abstracts. The remaining literature underwent a comprehensive assessment based on the inclusion and exclusion criteria. Ultimately, 85 articles met the criteria for inclusion. You can refer to Figure 1 for a detailed overview of the screening process.

### 3.1 Inclusion studies and characteristics

The 85 included studies were all from China and involved a total of 6,580 patients. Among these, 3,307 were in the experimental group, and 3,273 were in the control group. There were 11 CHIs involved, which included Aidi Injection plus DP (20 articles), Shenqi Fuzheng Injection plus DP (11 articles), Shenfu Injection plus DP (5 articles), Compound Kushen Injection plus DP (12 articles), Kangai Injection plus DP (14 articles), Brucea Javanica Oil Milk Injection plus DP (5 articles), Shenmai Injection plus DP (3 articles), Cinobufacini Injection plus DP (2 articles), Xiaoaiping Injection plus DP (2 articles), Kanglaite Injection plus DP (10 articles), and Astragalus Injection combined with DP (1 article). The basic characteristics of the included literature are presented in Table 1.

**TABLE 1:** Baseline characteristics.

Study ID	Sample size (E/C)	Sex (M/F)	Age (year, E/C)	Intervention in experimental group (WM + CHIs)^*^	Intervention in control group (WM/WM + another CHIs)^*^	Course of treatment (Days)	Outcomes
Bian et al. (2006)	34/30	44/20	30–75	AD×50 mL + DP	DP	28D×2	①②③
Zhu and Chen (2006)	30/30	32/28	36–74/33–74	AD×50 mL + DP	DP	21D×3	①②③
Chen et al. (2007)	32/32	39/25	47–72/49–71	AD×50 mL + DP	DP	21D×2	①②③
Yu and Li (2007)	30/30	44/16	51–77/50–78	SQ×250 mL + DP	DP	21D×2–3	①③
Lin (2008)	30/30	41/19	35–72/37–73	AD×50 mL + DP	DP	21D×2	①②③
Liu et al. (2008)	35/35	37/33	42–72/44–75	SF×40–60 mL + DP	DP	--	④⑤⑥⑦
Wang et al. (2008)	40/40	51/29	30–70	AD×80–100 mL + DP	DP	21D×2	①②③
Yang and Cao (2008)	50/55	65/40	42–75/43–72	KS×20 mL + DP	DP	14D×2	①②③
Zou and Wang (2008)	26/25	39/12	31–73/36–77	KA×40–50+DP	DP	21D×2	①②
Wang et al. (2009)	44/30	53/21	34–73/28–68	KS×20+DP	DP	21D×2	①③④⑤⑥⑦
Zhao (2009)	38/37	47/28	46–70/48–69	KA×60 mL + TP	DP	21D×2	①②③
Cui et al. (2010)	25/25	36/14	58.2/57.6	YDZ×20–30 mL + DP	DP	21D×2	①②③
Ding and Lu (2010)	25/25	33/17	42–73/44–76	KS×30 mL + DP	DP	21D×2	①②
Yao et al. (2010)	40/38	57/21	56–78/51–76	KS×40 mL + DP	DP	14D×4	①③④⑤⑥⑦
Zhang (2010)	15/15	17/13	45–70/43–73	KA×30 mL + DP	DP	30D×2	①②③
Zhen et al. (2010)	30/30	45/15	42–80/35–83	SM×60 mL + DP	DP	21D×2	①③
Zhu and Zhang (2010)	50/50	68/32	35–72/37–73	KLT×100 mL + DP	DP	21D×2	①②③
Du (2011)	60/60	94/26	42–71/46–68	AD×40 mL + DP	DP	28D×2	①②③
Jiang et al. (2011)	35/30	44/21	50–78/53–79	KA×40 mL + DP	DP	21D	①②③
Lin et al. (2011)	42/40	52/30	32–79/33–78	AD×50 mL + DP	DP	21D×2	①②③
Liu and Gao (2011)	50/50	51/49	57.1 ± 5.3/56.8 ± 6.2	SQ×250 mL + DP	DP	21D×2	②
Niu et al. (2011)	24/24	37/11	37–74/35–75	YDZ×30 mL + DP	DP	21D×2	①②③
Tang et al. (2011)	25/25	28/22	37–74	AD×50 mL + DP	DP	21–28D×2	①②③
Wang (2011)	49/49	65/33	30–78/32–76	AD×80–100 mL + DP	DP	21D×2	①②③
Wang et al. (2011)	24/28	37/15	56.6 ± 10.317/57.13 ± 11.079	SQ×250 mL + DP	DP	21D×2	①②③
Yang (2011)	30/30	41/19	35–72/37–73	AD×50 mL + DP	DP	21D×2	①②③
Chen et al. (2012)	34/34	46/22	40–76	KS×10 mL + DP	DP	--	①②③
Jiang (2012)	23/23	38/8	43–70	AD×50 mL + DP	DP	21D×2	①③
Tu (2012)	32/31	40/23	70–85	KA×40 mL + DP	DP	21D×2	①②③
Yu et al. (2012)	32/32	39/25	47–72/49–71	HCS×20 mL + DP	DP	21D×2	①②③
Ge and Luo (2013)	41/39	52/28	55–77/53–76	AD×50 mL + DP	DP	28D	①⑤⑥⑦
Ma (2013)	28/28	35/21	65–81/67–83	SQ×250 mL + DP	DP	21D×3	①②③
Wang and Xu (2013)	46/46	61/31	41–73/43–75	KS×20 mL + DP	DP	21D×4	①②③
Wei (2013)	32/32	42/22	37–65	KA×40 mL + DP	DP	21D×2	①②③⑤⑥⑦
Zhang (2013)	37/37	51/23	38–75	YDZ×30 mL + DP	DP	21D×4	①③
Bai et al. (2014)	39/39	41/37	36–77/42–75	SF×80 mL + DP	DP	21D×2	①②③
Shan et al. (2014)	40/40	44/36	41–76	SQ×250 mL + DP	DP	21D×2	①②④⑤⑥⑦
Deng et al. (2014)	34/34	42/26	63.88 ± 1.99/64.29 ± 2.07	KLT×200 mL + DP	DP	21D×2	④⑤⑥⑦
Gao and Zhang (2014)	36/35	37/34	--	AD×50 mL + DP	DP	21D×3	①②
He et al. (2014)	60/60	94/26	46–68/42–71	AD×40 mL + DP	DP	28D×2	①②③
Liu et al. (2014)	51/51	59/42	62/64	KA×40 mL + DP	DP	21D×2	③④⑤⑦
Ning (2014)	45/45	47/43	29–71	KA×50 mL + DP	DP	21D×2	①③
Wu et al. (2014)	36/32	43/25	38–72	SQ×250 mL + DP	DP	21D×2	①②③④⑤⑥⑦
Zhang and Liu (2014)	45/45	61/29	54–80/55–82	YDZ×40 mL + DP	DP	21D×4	①③④⑤⑥⑦
Cai et al. (2015)	56/56	64/48	42–78	YDZ×30 mL + DP	DP	28D×2	①②③
Han et al. (2015)	28/28	31/25	58.24 ± 8.31/55.17 ± 8.43	SQ×250+DP	DP	28D×3	①②③④⑤⑦
Huang et al. (2015)	60/60	82/38	42–79	KS×30 mL + DP	DP	21D×2	①③
Mo (2015)	43/43	49/37	57.28 ± 6.32/56.96 ± 6.17	AD×50 mL + DP	DP	21D×2	①②③
Shen et al. (2015)	28/28	39/17	50–75	XAP×40–60+DP	DP	21D×2	①②③
Xie et al. (2015)	40/40	53/27	20–72/39–75	SF×80 mL + DP	DP	28D×2	③
Ai et al. (2016)	68/68	54/82	34–72/38–71	KS×20 mL + DP	DP	21D×2	①③
Cao et al. (2016)	40/40	49/31	57.41 ± 9.04/57.15 ± 9.24	HCS×10–20 mL + DP	DP	28D	①③
Dong (2016)	40/39	36/43	18–78	KLT×200 mL + DP	DP	21D×2	①②③④⑤⑦
Xu et al. (2016)	30/30	41/19	48–73/47–74	SQ×250+DP	DP	21D×4	①②
Liang et al. (2016)	39/39	--	--	SF×80 mL + DP	DP	21D×2	④⑤⑥⑦
Lu et al. (2016)	24/24	28/20	44–71	KLT×200 mL + DP	DP	21D×2	①③④⑤⑥⑦
Luo (2016)	21/21	25/17	36–65/40–68	SQ×250 mL + DP	DP	21D×4	①②③
Sun and Lu (2016)	84/84	106/64	32–71/34–77	KA×20 mL + DP	DP	21D×3	①②③
Wang et al. (2016)	23/23	--	40–70	AD×50 mL + DP	DP	21D×2	①③
Wang (2016)	37/36	53/20	58.79 ± 8.06	AD×100 mL + DP	DP	21D×2	③
Zhen et al. (2016)	32/32	39/25	46–71/47–72	KLT×200 mL + DP	DP	21D×2	④⑤⑥⑦
Bai et al. (2017)	39/39	41/37	56.42 ± 11.81/54.25 ± 13.62	SF×80 mL + DP	DP	21D×2	③
Bao and Jiang (2017)	49/49	62/36	34–75/33–77	AD×80 mL + DP	DP	21D×2	①②③
Chen (2017)	58/57	75/40	59–73/65–78	KA×60 mL + DP	DP	21D×4	①③
Cheng et al. (2017)	30/30	27/33	40–75/41–72	SM×50 mL + DP	DP	-	①②
Ren et al. (2017)	26/26	36/16	61–83/62–85	KS×20 mL + DP	DP	21D×3	①②③
Tian and Yang (2017)	42/42	53/31	53–86/54–87	KLT×200 mL + DP	DP	21D×2	①④⑤⑥⑦
Ye and Fu (2017)	30/30	38/22	42–62	XAP×60 mL + DP	DP	21D	①②③
Chen et al. (2018b)	48/48	52/44	44–67/42–68	SM×100 mL + DP	DP	21D×4	①②
Chen et al. (2018a)	68/68	90/46	36–71/40–73	KS×20 mL + DP	DP	21D×2	①②③
Fu et al. (2018)	50/50	60/40	42–71/34–69	KA×40 mL + DP	DP	21D×2	①②③
Jia (2018)	31/31	34/28	43–74/44–72	KLT×200 mL + DP	DP	21D×2	①④⑤⑥⑦
Wang et al. (2018)	38/37	44/31	39–73/40–72	HQ×40 mL + DP	DP	21D×4	①④⑤⑥⑦
Yu et al. (2018)	40/40	51/29	45–74/43–73	KA×40 mL + DP	DP	21D×2	①③
Zhang (2018)	35/35	38/32	61–71/63–75	KA×40 mL + DP	DP	21D	①④⑤⑥
Dai (2019)	40/40	43/37	34–78/32–76	AD×80–90 mL + DP	DP	21D×2	①②③
Gao (2019)	30/30	32/28	39–69/36–70	KA×40 mL + DP	DP	21D×2	①③
Gong et al. (2019)	42/42	59/25	58.31 ± 7.60/56.62 ± 8.10	SQ×250 mL + DP	DP	21D×4	①③④⑤⑥⑦
Zhao (2019)	15/15	16/14	69–78/62–82	KLT×100 mL + DP	DP	21D	①③
Zhen (2020)	50/50	54/46	55–74/54–76	KLT×100 mL + DP	DP	21D×4	①
Zhu and Wu (2020)	40/40	45/35	47–71/45–69	SQ×250 mL + DP	DP	28D×3	①③④⑤
Fan and Liu (2021)	59/59	67/51	43–81/44–79	KS×20 mL + DP	DP	21D×3	①
Liu et al. (2021)	48/48	58/38	49–79/47–76	KS×20 mL + DP	DP	21D×4	①③⑤⑦
Zhou et al. (2021)	40/40	53/27	54–78/59–79	KLT×200 mL + DP	DP	21D×2	①
Shen and Na (2022)	60/60	68/52	51–75	AD×50+DP	DP	21D	①④⑤⑥⑦

Note: * The groups received the same treatment regimens of WM; E/C, experimental group/control group; M/F, male/female; CHIs, Chinese herbal injections; WM, western medicine; AD, aidi injection; SQ, shenqifuzheng injection; SF, shenfu injection; KS, compound kushen injection; KA, kangai injection; YDZ, brucea javanica oil milk injection; SM, shenmai injection; HCS, cinobufacini injection; XAP, xiaoaiping injection; KLT, kanglaite injection; HQ, Astragalus injection; ①, Response rate; ②, QoL; ③, Adverse drug reactions; ④, Anti-CD3^+^; ⑤, Anti-CD4^+^; ⑥, Anti-CD8^+^; ⑦,Anti-CD4+/Anti-CD8^+^.

### 3.2 Methodological quality

**FIGURE 2:**
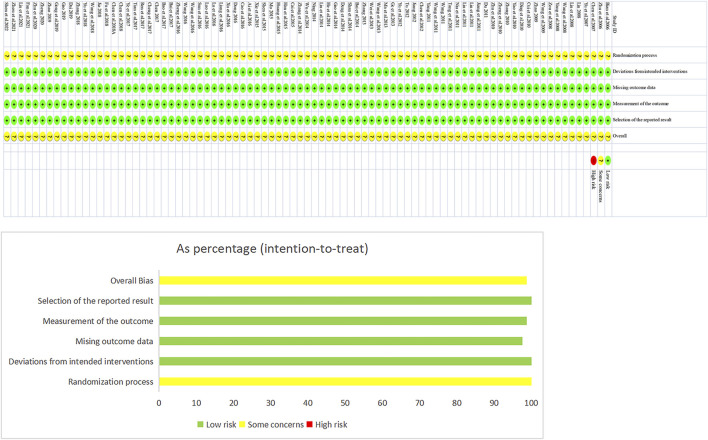
Assessment Figure of risk of bias.

The risk-of-bias assessment results for the 85 included studies are shown in Figure 2. In terms of bias in randomization, all 85 studies were assessed as having a potential risk due to the lack of a description of randomization and allocation concealment. All studies were assessed as having a low risk of bias in terms of deviations from established interventions, missing data on outcomes, measurements, and selective reporting. No other sources of bias were found in any of the included studies, which were assessed to be at low risk. Taken together, the risk of bias in the included literature was low.

### 3.3 Network analysis results

#### 3.3.1 Network diagram

**FIGURE 3:**
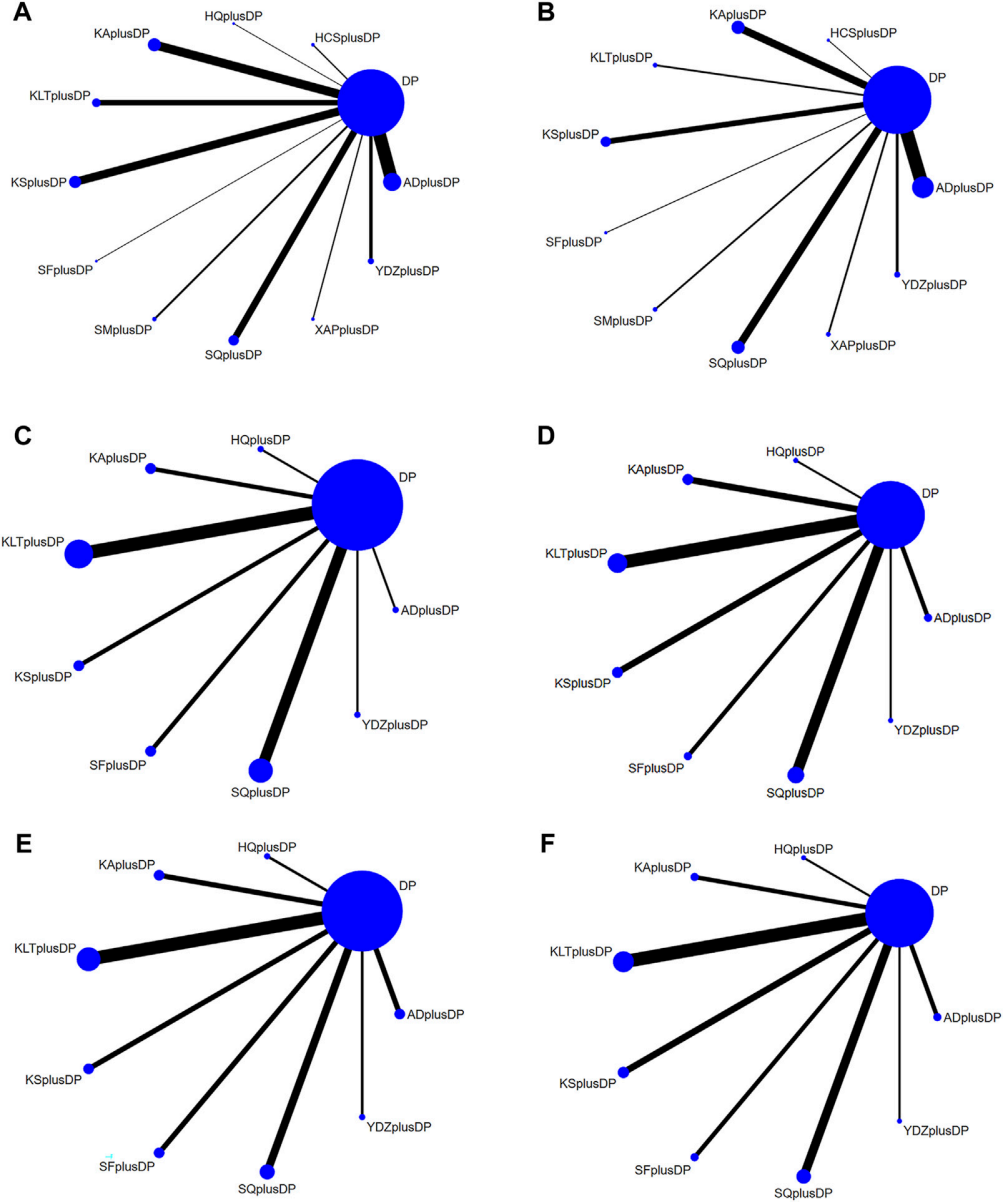
Network Diagram. **(A)** Response rate network structure; **(B)** QoL network structure; **(C)** CD3^+^ network structure; **(D)** CD4^+^ network structure; **(E)** CD8^+^ network structure; **(F)** CD4^+^/CD8^+^ network structure.

The 85 included studies (Bian et al., 2006; Zhu and Chen, 2006; Chen et al., 2007; Yu and Li, 2007; Lin, 2008; Liu et al., 2008; Wang et al., 2008; Yang and Cao, 2008; Zou and Wang, 2008; Wang et al., 2009; Zhao, 2009; Cui et al., 2010; Ding and Lu, 2010; Yao et al., 2010; Zhang, 2010; Zhen et al., 2010; Zhu and Zhang, 2010; Du, 2011; Jiang et al., 2011; Lin et al., 2011; Liu and Gao, 2011; Niu et al., 2011; Tang et al., 2011; Wang, 2011; Wang et al., 2011; Yang, 2011; Chen et al., 2012; Jiang, 2012; Tu, 2012; Yu et al., 2012; Ge and Luo, 2013; Ma, 2013; Wang and Xu, 2013; Wei, 2013; Zhang, 2013; Bai et al., 2014; Shan et al., 2014; Deng et al., 2014; Gao and Zhang, 2014; He et al., 2014; Liu et al., 2014; Ning, 2014; Wu et al., 2014; Zhang and Liu, 2014; Cai et al., 2015; Han et al., 2015; Huang et al., 2015; Mo, 2015; Shen et al., 2015; Xie et al., 2015; Ai et al., 2016; Cao et al., 2016; Dong, 2016; Xu et al., 2016; Liang et al., 2016; Lu et al., 2016; Luo, 2016; Sun and Lu, 2016; Wang et al., 2016; Wang, 2016; Zhen et al., 2016; Bai et al., 2017; Bao and Jiang, 2017; Chen, 2017; Cheng et al., 2017; Ren et al., 2017; Tian and Yang, 2017; Ye and Fu, 2017; Chen et al., 2018b; Chen et al., 2018a; Fu et al., 2018; Jia, 2018; Wang et al., 2018; Yu et al., 2018; Zhang, 2018; Dai, 2019; Gao, 2019; Gong et al., 2019; Zhao, 2019; Zhen, 2020; Zhu and Wu, 2020; Fan and Liu, 2021; Liu et al., 2021; Zhou et al., 2021; Shen and Na, 2022) covered 11 different CHI interventions: Aidi Injection (20 RCTs), Astragalus Injection (1 RCT), Compound Kushen Injection (12 RCTs), Kangai Injection (14 RCTs), Kanglaite Injection (10 RCTs), Shenfu Injection (5 RCTs), Shenmai Injection (3 RCTs), Shenqi Fuzheng Injection (11 RCTs), Xiaoaiping Injection (2 RCTs), Brucea Javanica Oil Milk Injection (5 RCTs), and Cinobufacini Injection (2 RCTs). The network structure between these interventions is depicted in Figure 3. In the figure, the thickness of the lines corresponds to the volume of literature involved in the pairwise comparisons, and the size of the circles’ diameter is proportional to the number of participants included in each intervention.

#### 3.3.2 Response rate

**FIGURE 4:**
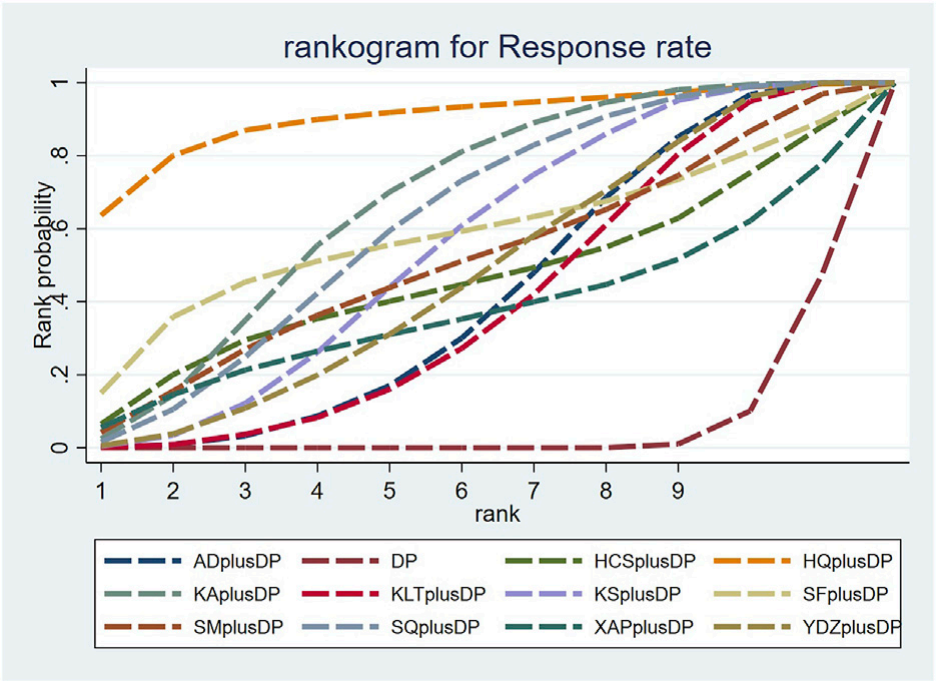
Cumulative probability line chart of response rate.

Seventy-six studies reported information on the response rate. The results indicated that seven regimens, including Aidi Injection plus DP (RR = 1.24, 95% CI: 1.12, 1.38), Astragalus Injection plus DP (RR = 1.77, 95% CI: 1.15, 2.92), Kangai Injection plus DP (RR = 1.35, 95% CI: 1.19, 1.55), Kanglaite Injection plus DP (RR = 1.23, 95% CI: 1.10, 1.39), Compound Kushen Injection plus DP (RR = 1.29, 95% CI: 1.16, 1.45), Shenqi Fuzheng Injection plus DP (RR = 1.33, 95% CI: 1.15, 1.55), and Brucea Javanica Oil Milk Injection plus DP (RR = 1.26, 95% CI: 1.09, 1.48), were superior to DP alone in improving the response rate, and the differences were statistically significant. Other interventions did not show significant differences in pairwise comparisons (see Table 2). According to the cumulative probability results, Astragalus Injection plus DP (SUCRAS: 90.25%), Kangai Injection plus DP (SUCRAS: 67.26%), and Shenqi Fuzheng Injection plus DP (SUCRAS: 61.90%) were likely to be the top three effective regimens in improving the response rate (see Figure 4).

**TABLE 2:** Response rate league table.

ADplusDP	0.81 (0.73, 0.89)	1 (0.64, 1.54)	1.43 (0.91, 2.4)	1.09 (0.93, 1.29)	0.99 (0.86, 1.16)	1.04 (0.9, 1.21)	1.09 (0.65, 1.85)	1.03 (0.76, 1.4)	1.07 (0.9, 1.3)	0.95 (0.6, 1.57)	1.02 (0.85, 1.24)
1.24 (1.12, 1.38)	DP	1.24 (0.81, 1.92)	**1.77 (1.15, 2.92)**	**1.35 (1.19, 1.55)**	**1.23 (1.1, 1.39)**	**1.29 (1.16, 1.45)**	1.35 (0.81, 2.27)	1.28 (0.96, 1.7)	**1.33 (1.15, 1.55)**	1.17 (0.74, 1.91)	**1.26 (1.09, 1.48)**
1 (0.65, 1.55)	0.81 (0.52, 1.23)	HCSplusDP	1.43 (0.75, 2.74)	1.1 (0.7, 1.71)	0.99 (0.64, 1.54)	1.04 (0.66, 1.62)	1.08 (0.55, 2.11)	1.03 (0.63, 1.71)	1.06 (0.69, 1.66)	0.94 (0.5, 1.86)	1.02 (0.65, 1.61)
0.7 (0.42, 1.1)	0.56 (0.34, 0.87)	0.7 (0.37, 1.33)	HQplusDP	0.76 (0.46, 1.21)	0.69 (0.41, 1.09)	0.73 (0.43, 1.14)	0.76 (0.38, 1.5)	0.72 (0.41, 1.22)	0.75 (0.45, 1.18)	0.66 (0.34, 1.29)	0.71 (0.42, 1.13)
0.92 (0.77, 1.08)	0.74 (0.65, 0.84)	0.91 (0.59, 1.44)	1.31 (0.83, 2.2)	KAplusDP	0.91 (0.76, 1.08)	0.95 (0.8, 1.14)	1 (0.59, 1.71)	0.95 (0.68, 1.28)	0.98 (0.8, 1.2)	0.87 (0.53, 1.44)	0.93 (0.76, 1.15)
1.01 (0.86, 1.17)	0.82 (0.72, 0.91)	1.01 (0.65, 1.57)	1.44 (0.92, 2.44)	1.1 (0.93, 1.31)	KLTplusDP	1.05 (0.9, 1.23)	1.1 (0.65, 1.87)	1.04 (0.77, 1.41)	1.08 (0.89, 1.32)	0.95 (0.6, 1.57)	1.03 (0.85, 1.24)
0.96 (0.83, 1.12)	0.77 (0.69, 0.86)	0.96 (0.62, 1.52)	1.37 (0.88, 2.32)	1.05 (0.88, 1.24)	0.95 (0.81, 1.12)	KSplusDP	1.04 (0.62, 1.77)	0.99 (0.72, 1.35)	1.02 (0.86, 1.25)	0.91 (0.57, 1.5)	0.97 (0.81, 1.19)
0.92 (0.54, 1.54)	0.74 (0.44, 1.24)	0.92 (0.48, 1.81)	1.32 (0.67, 2.6)	1 (0.58, 1.71)	0.91 (0.53, 1.54)	0.96 (0.56, 1.62)	SFplusDP	0.95 (0.52, 1.75)	0.98 (0.58, 1.7)	0.87 (0.43, 1.79)	0.93 (0.55, 1.61)
0.97 (0.71, 1.32)	0.78 (0.59, 1.05)	0.97 (0.59, 1.59)	1.4 (0.82, 2.44)	1.05 (0.78, 1.47)	0.96 (0.71, 1.3)	1.01 (0.74, 1.4)	1.06 (0.57, 1.93)	SMplusDP	1.04 (0.76, 1.43)	0.91 (0.54, 1.64)	0.99 (0.72, 1.36)
0.94 (0.77, 1.11)	0.75 (0.65, 0.87)	0.94 (0.6, 1.45)	1.34 (0.85, 2.22)	1.02 (0.83, 1.24)	0.93 (0.76, 1.12)	0.98 (0.8, 1.17)	1.02 (0.59, 1.73)	0.96 (0.7, 1.31)	SQplusDP	0.88 (0.55, 1.49)	0.95 (0.78, 1.17)
1.06 (0.64, 1.68)	0.85 (0.52, 1.35)	1.07 (0.54, 2.01)	1.52 (0.77, 2.92)	1.15 (0.7, 1.89)	1.05 (0.64, 1.68)	1.1 (0.66, 1.77)	1.15 (0.56, 2.35)	1.09 (0.61, 1.84)	1.14 (0.67, 1.83)	XAPplusDP	1.08 (0.65, 1.73)
0.98 (0.81, 1.17)	0.8 (0.67, 0.92)	0.98 (0.62, 1.53)	1.41 (0.88, 2.37)	1.07 (0.87, 1.31)	0.98 (0.8, 1.18)	1.03 (0.84, 1.23)	1.08 (0.62, 1.83)	1.01 (0.73, 1.39)	1.05 (0.85, 1.29)	0.93 (0.58, 1.55)	YDZplusDP

The bold values indicated that seven regimens, including Aidi Injection plus DP (RR = 1.24, 95% CI: 1.12, 1.38), Astragalus Injection plus DP (RR = 1.77, 95% CI: 1.15, 2.92), Kangai Injection plus DP (RR = 1.35, 95% CI: 1.19, 1.55), Kanglaite Injection plus DP (RR = 1.23, 95% CI: 1.10, 1.39), Compound Kushen Injection plus DP (RR = 1.29, 95% CI: 1.16, 1.45), Shenqi Fuzheng Injection plus DP (RR = 1.33, 95% CI: 1.15, 1.55), and Brucea Javanica Oil Milk Injection plus DP (RR = 1.26, 95% CI: 1.09, 1.48), were superior to DP alone in improving the response rate.

#### 3.3.3 QoL

**FIGURE 5:**
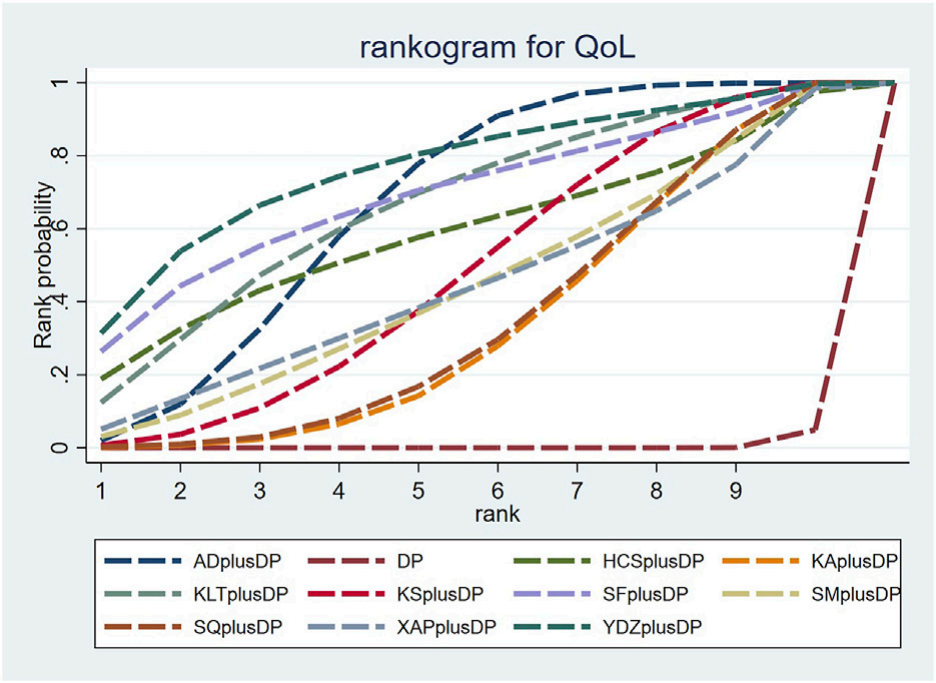
Cumulative probability line chart of quality of life.

Forty-seven studies reported information on Quality of Life (QoL). The results indicated that ten regimens, including Aidi Injection plus DP (RR = 1.84, 95% CI: 1.60, 2.14), Cinobufacini Injection plus DP (RR = 1.83, 95% CI: 1.00, 3.78), Kangai Injection plus DP (RR = 1.55, 95% CI: 1.30, 1.88), Kanglaite Injection plus DP (RR = 1.90, 95% CI: 1.32, 2.80), Compound Kushen Injection plus DP (RR = 1.67, 95% CI: 1.37, 2.08), Shenfu Injection plus DP (RR = 2.00, 95% CI: 1.17, 3.95), Shenmai Injection plus DP (RR = 1.62, 95% CI: 1.16, 2.40), Shenqi Fuzheng Injection plus DP (RR = 1.56, 95% CI: 1.29, 1.91), Xiaoaiping Injection plus DP (RR = 1.62, 95% CI: 1.05, 2.57), and Brucea Javanica Oil Milk Injection plus DP (RR = 2.12, 95% CI: 1.25, 3.68), were superior to DP alone in improving QoL, and the differences were statistically significant. Other interventions did not show significant differences in pairwise comparisons (see Table 3). According to the cumulative probability results, Brucea Javanica Oil Milk Injection plus DP (SUCRAs: 76.89%), Shenfu Injection plus DP (SUCRAs: 69.49%), and Aidi Injection plus DP (SUCRAs: 66.94%) were likely to be the top three effective regimens in improving QoL (see Figure 5).

**TABLE 3:** QoL league table.

ADplusDP	0.54 (0.47, 0.62)	0.99 (0.54, 2.07)	0.84 (0.67, 1.07)	1.03 (0.69, 1.56)	0.91 (0.71, 1.17)	1.08 (0.62, 2.17)	0.88 (0.61, 1.33)	0.84 (0.66, 1.08)	0.88 (0.56, 1.42)	1.16 (0.66, 2.02)
1.84 (1.6, 2.14)	DP	**1.83 (1, 3.78)**	**1.55 (1.3, 1.88)**	**1.9 (1.32, 2.8)**	**1.67 (1.37, 2.08)**	**2 (1.17, 3.95)**	**1.62 (1.16, 2.4)**	**1.56 (1.29, 1.91)**	**1.62 (1.05, 2.57)**	**2.12 (1.25, 3.68)**
1.01 (0.48, 1.84)	0.55 (0.26, 1)	HCSplusDP	0.85 (0.41, 1.58)	1.04 (0.47, 2.1)	0.91 (0.43, 1.72)	1.1 (0.45, 2.72)	0.89 (0.4, 1.8)	0.85 (0.4, 1.61)	0.88 (0.4, 1.87)	1.17 (0.47, 2.64)
1.19 (0.94, 1.49)	0.64 (0.53, 0.77)	1.18 (0.63, 2.45)	KAplusDP	1.22 (0.81, 1.88)	1.07 (0.82, 1.43)	1.29 (0.73, 2.52)	1.05 (0.71, 1.59)	1 (0.77, 1.31)	1.04 (0.64, 1.69)	1.37 (0.78, 2.43)
0.97 (0.64, 1.44)	0.53 (0.36, 0.76)	0.96 (0.48, 2.14)	0.82 (0.53, 1.24)	KLTplusDP	0.88 (0.57, 1.34)	1.06 (0.54, 2.23)	0.86 (0.51, 1.46)	0.82 (0.53, 1.25)	0.85 (0.48, 1.53)	1.12 (0.6, 2.19)
1.1 (0.85, 1.41)	0.6 (0.48, 0.73)	1.09 (0.58, 2.32)	0.93 (0.7, 1.22)	1.14 (0.74, 1.76)	KSplusDP	1.19 (0.67, 2.43)	0.97 (0.65, 1.5)	0.93 (0.7, 1.24)	0.97 (0.59, 1.61)	1.27 (0.74, 2.22)
0.92 (0.46, 1.61)	0.5 (0.25, 0.85)	0.91 (0.37, 2.24)	0.78 (0.4, 1.36)	0.94 (0.45, 1.86)	0.84 (0.41, 1.5)	SFplusDP	0.81 (0.38, 1.57)	0.78 (0.39, 1.4)	0.8 (0.36, 1.67)	1.06 (0.46, 2.28)
1.13 (0.75, 1.64)	0.62 (0.42, 0.86)	1.12 (0.56, 2.52)	0.95 (0.63, 1.4)	1.17 (0.68, 1.95)	1.03 (0.67, 1.53)	1.23 (0.64, 2.64)	SMplusDP	0.96 (0.62, 1.43)	1 (0.57, 1.74)	1.3 (0.71, 2.47)
1.18 (0.93, 1.51)	0.64 (0.52, 0.77)	1.18 (0.62, 2.5)	1 (0.76, 1.3)	1.22 (0.8, 1.88)	1.07 (0.81, 1.42)	1.29 (0.71, 2.55)	1.04 (0.7, 1.61)	SQplusDP	1.04 (0.65, 1.73)	1.36 (0.79, 2.46)
1.14 (0.7, 1.8)	0.62 (0.39, 0.95)	1.13 (0.54, 2.51)	0.96 (0.59, 1.56)	1.17 (0.65, 2.09)	1.04 (0.62, 1.69)	1.24 (0.6, 2.79)	1 (0.57, 1.76)	0.96 (0.58, 1.54)	XAPplusDP	1.31 (0.63, 2.63)
0.87 (0.49, 1.52)	0.47 (0.27, 0.8)	0.86 (0.38, 2.13)	0.73 (0.41, 1.28)	0.89 (0.46, 1.67)	0.79 (0.45, 1.35)	0.94 (0.44, 2.19)	0.77 (0.41, 1.4)	0.74 (0.41, 1.26)	0.76 (0.38, 1.58)	YDZplusDP

The bold values indicated that ten regimens, including Aidi Injection plus DP (RR = 1.84, 95% CI: 1.60, 2.14), Cinobufacini Injection plus DP (RR = 1.83, 95% CI: 1.00, 3.78), Kangai Injection plus DP (RR = 1.55, 95% CI: 1.30, 1.88), Kanglaite Injection plus DP (RR = 1.90, 95% CI: 1.32, 2.80), Compound Kushen Injection plus DP (RR = 1.67, 95% CI: 1.37, 2.08), Shenfu Injection plus DP (RR = 2.00, 95% CI: 1.17, 3.95), Shenmai Injection plus DP (RR = 1.62, 95% CI: 1.16, 2.40), Shenqi Fuzheng Injection plus DP (RR = 1.56, 95% CI: 1.29, 1.91), Xiaoaiping Injection plus DP (RR = 1.62, 95% CI: 1.05, 2.57), and Brucea Javanica Oil Milk Injection plus DP (RR = 2.12, 95% CI: 1.25, 3.68), were superior to DP alone in improving QoL.

#### 3.3.4 CD3^+^

**FIGURE 6:**
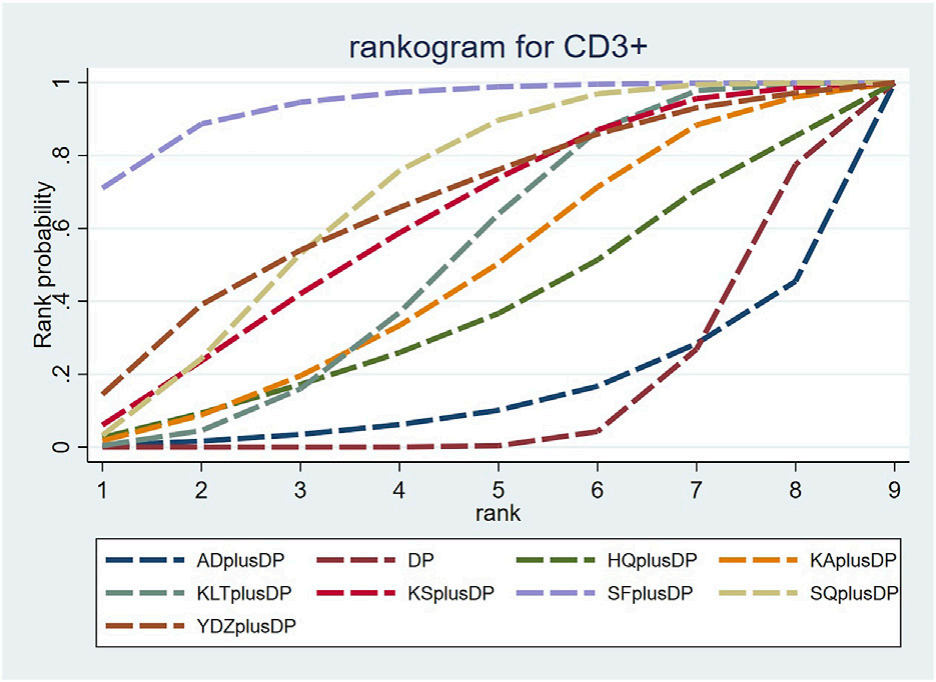
Cumulative probability line chart of CD3^+^.

Twenty studies reported information on CD3^+^ levels. The results indicated that three regimens, including Kanglaite Injection plus DP (MD = 7.41, 95% CI: 1.50, 13.27), Shenfu Injection plus DP (MD = 18.79, 95% CI: 8.36, 29.49), and Shenqi Fuzheng Injection plus DP (MD = 10.51, 95% CI: 4.11, 16.99), were more effective than DP alone in improving CD3^+^ levels, and the differences were statistically significant. Shenfu Injection plus DP (MD = 20.86, 95% CI: 3.38, 38.80) was found to be more effective in improving CD3^+^ levels compared to Aidi Injection plus DP. Other interventions did not show significant differences in pairwise comparisons (see Table 4). According to the cumulative probability results, Shenfu Injection plus DP (SUCRAs: 93.75%), Shenqi Fuzheng Injection plus DP (SUCRAs: 67.81%), and Brucea Javanica Oil Milk Injection plus DP (SUCRAs: 65.69%) were likely to be the top three approaches for improving CD3^+^ levels (see Figure 6).

**TABLE 4:** CD3^+^ league table.

ADplusDP	2.05 (−12.18, 16.32)	6.37 (−13.77, 26.58)	8.52 (−9.02, 26.06)	9.49 (−5.92, 24.92)	11.44 (−6.01, 29.72)	20.86 (3.38, 38.8)	12.61 (−3.07, 28.23)	12.98 (−7.38, 33.37)
−2.05 (−16.32, 12.18)	DP	4.31 (−10.01, 18.62)	6.45 (−3.85, 16.61)	**7.41 (1.5, 13.27)**	9.36 (−0.89, 20.53)	**18.79 (8.36, 29.49)**	**10.51 (4.11, 16.99)**	10.9 (−3.57, 25.56)
−6.37 (−26.58, 13.77)	−4.31 (−18.62, 10.01)	HQplusDP	2.14 (−15.44, 19.67)	3.11 (−12.3, 18.58)	5.04 (−12.42, 23.3)	14.48 (−3.11, 32.34)	6.21 (−9.41, 21.91)	6.58 (−13.75, 27.07)
−8.52 (−26.06, 9.02)	−6.45 (−16.61, 3.85)	−2.14 (−19.67, 15.44)	KAplusDP	0.97 (−10.83, 12.79)	2.91 (−11.43, 18.19)	12.34 (−2.26, 27.19)	4.08 (−7.97, 16.21)	4.45 (−13.23, 22.26)
−9.49 (−24.92, 5.92)	−7.41 (−13.27, −1.5)	−3.11 (−18.58, 12.3)	−0.97 (−12.79, 10.83)	KLTplusDP	1.95 (−9.83, 14.59)	11.37 (−0.58, 23.62)	3.1 (−5.54, 11.84)	3.48 (−12.18, 19.25)
−11.44 (−29.72, 6.01)	−9.36 (−20.53, 0.89)	−5.04 (−23.3, 12.42)	−2.91 (−18.19, 11.43)	−1.95 (−14.59, 9.83)	KSplusDP	9.43 (−5.89, 24.18)	1.15 (−11.78, 13.16)	1.52 (−16.89, 19.22)
−20.86 (−38.8, −3.38)	−18.79 (−29.49, −8.36)	−14.48 (−32.34, 3.11)	−12.34 (−27.19, 2.26)	−11.37 (−23.62, 0.58)	−9.43 (−24.18, 5.89)	SFplusDP	−8.27 (−20.8, 3.95)	−7.88 (−25.99, 10)
−12.61 (−28.23, 3.07)	−10.51 (−16.99, −4.11)	−6.21 (−21.91, 9.41)	−4.08 (−16.21, 7.97)	−3.1 (−11.84, 5.54)	−1.15 (−13.16, 11.78)	8.27 (−3.95, 20.8)	SQplusDP	0.38 (−15.51, 16.34)
−12.98 (−33.37, 7.38)	−10.9 (−25.56, 3.57)	−6.58 (−27.07, 13.75)	−4.45 (−22.26, 13.23)	−3.48 (−19.25, 12.18)	−1.52 (−19.22, 16.89)	7.88 (−10, 25.99)	−0.38 (−16.34, 15.51)	YDZplusDP

The bold values indicated that three regimens, including Kanglaite Injection plus DP (MD = 7.41, 95% CI: 1.50, 13.27), Shenfu Injection plus DP (MD = 18.79, 95% CI: 8.36, 29.49), and Shenqi Fuzheng Injection plus DP (MD = 10.51, 95% CI: 4.11, 16.99), were more effective than DP alone in improving CD3^+^ levels.

#### 3.4.5 CD4^+^

**FIGURE 7:**
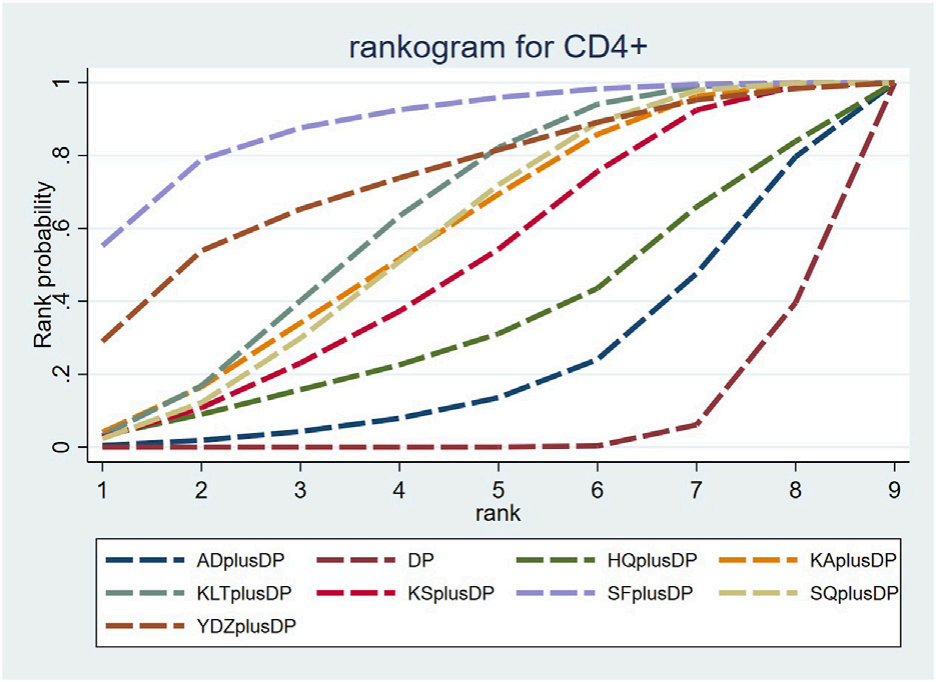
Cumulative probability line chart of CD4^+^.

Twenty-three studies reported information on CD4^+^ levels. The results indicated that six regimens, including Kangai Injection plus DP (MD = 6.15, 95% CI: 1.57, 10.75), Kanglaite Injection plus DP (MD = 6.59, 95% CI: 3.35, 9.89), Compound Kushen Injection plus DP (MD = 5.36, 95% CI: 0.58, 10.37), Shenfu Injection plus DP (MD = 10.38, 95% CI: 4.33, 17.11), Shenqi Fuzheng Injection plus DP (MD = 6.10, 95% CI: 2.52, 9.74), and Brucea Javanica Oil Milk Injection plus DP (MD = 8.40, 95% CI: 0.28, 16.52), were significantly more effective than DP alone in improving CD4^+^ levels, and the differences were statistically significant. Other interventions did not show significant differences in pairwise comparisons (see Table 5). According to the cumulative probability results, Shenfu Injection plus DP (SUCRAs: 88.50%), Brucea Javanica Oil Milk Injection plus DP (SUCRAs: 73.28%), and Kanglaite Injection plus DP (SUCRAs: 62.35%) were likely to be the top three regimens in improving CD4^+^ levels (see Figure 7).

**TABLE 5:** CD4^+^ league table.

ADplusDP	−2.06 (−8.05, 3.71)	1.36 (−8.6, 11.11)	4.1 (−3.48, 11.48)	4.54 (−2.28, 11.21)	3.3 (−4.3, 10.96)	8.32 (−0.13, 17.21)	4.05 (−2.92, 10.89)	6.34 (−3.79, 16.29)
2.06 (−3.71, 8.05)	DP	3.41 (−4.51, 11.32)	**6.15 (1.57, 10.75)**	**6.59 (3.35, 9.89)**	**5.36 (0.58, 10.37)**	**10.38 (4.33, 17.11)**	**6.1 (2.52, 9.74)**	**8.4 (0.28, 16.52)**
−1.36 (−11.11, 8.6)	−3.41 (−11.32, 4.51)	HQplusDP	2.74 (−6.42, 11.88)	3.19 (−5.32, 11.8)	1.94 (−7.19, 11.38)	6.97 (−2.85, 17.49)	2.69 (−5.96, 11.4)	4.99 (−6.31, 16.36)
−4.1 (−11.48, 3.48)	−6.15 (−10.75, −1.57)	−2.74 (−11.88, 6.42)	KAplusDP	0.45 (−5.18, 6.09)	−0.79 (−7.38, 6.02)	4.22 (−3.25, 12.44)	−0.05 (−5.89, 5.8)	2.25 (−7.13, 11.56)
−4.54 (−11.21, 2.28)	−6.59 (−9.89, −3.35)	−3.19 (−11.8, 5.32)	−0.45 (−6.09, 5.18)	KLTplusDP	−1.24 (−7.03, 4.77)	3.78 (−3.13, 11.23)	−0.49 (−5.36, 4.39)	1.8 (−6.98, 10.48)
−3.3 (−10.96, 4.3)	−5.36 (−10.37, −0.58)	−1.94 (−11.38, 7.19)	0.79 (−6.02, 7.38)	1.24 (−4.77, 7.03)	KSplusDP	5.01 (−2.81, 13.23)	0.75 (−5.43, 6.71)	3.02 (−6.55, 12.43)
−8.32 (−17.21, 0.13)	−10.38 (−17.11, −4.33)	−6.97 (−17.49, 2.85)	−4.22 (−12.44, 3.25)	−3.78 (−11.23, 3.13)	−5.01 (−13.23, 2.81)	SFplusDP	−4.27 (−11.91, 2.77)	−1.96 (−12.73, 7.97)
−4.05 (−10.89, 2.92)	−6.1 (−9.74, −2.52)	−2.69 (−11.4, 5.96)	0.05 (−5.8, 5.89)	0.49 (−4.39, 5.36)	−0.75 (−6.71, 5.43)	4.27 (−2.77, 11.91)	SQplusDP	2.28 (−6.6, 11.11)
−6.34 (−16.29, 3.79)	−8.4 (−16.52, −0.28)	−4.99 (−16.36, 6.31)	−2.25 (−11.56, 7.13)	−1.8 (−10.48, 6.98)	−3.02 (−12.43, 6.55)	1.96 (−7.97, 12.73)	−2.28 (−11.11, 6.6)	YDZplusDP

The bold values indicated that six regimens, including Kangai Injection plus DP (MD = 6.15, 95% CI: 1.57, 10.75), Kanglaite Injection plus DP (MD = 6.59, 95% CI: 3.35, 9.89), Compound Kushen Injection plus DP (MD = 5.36, 95% CI: 0.58, 10.37), Shenfu Injection plus DP (MD = 10.38, 95% CI: 4.33, 17.11), Shenqi Fuzheng Injection plus DP (MD = 6.10, 95% CI: 2.52, 9.74), and Brucea Javanica Oil Milk Injection plus DP (MD = 8.40, 95% CI: 0.28, 16.52), were significantly more effective than DP alone in improving CD4^+^ levels.

#### 3.4.6 CD8^+^

**FIGURE 8:**
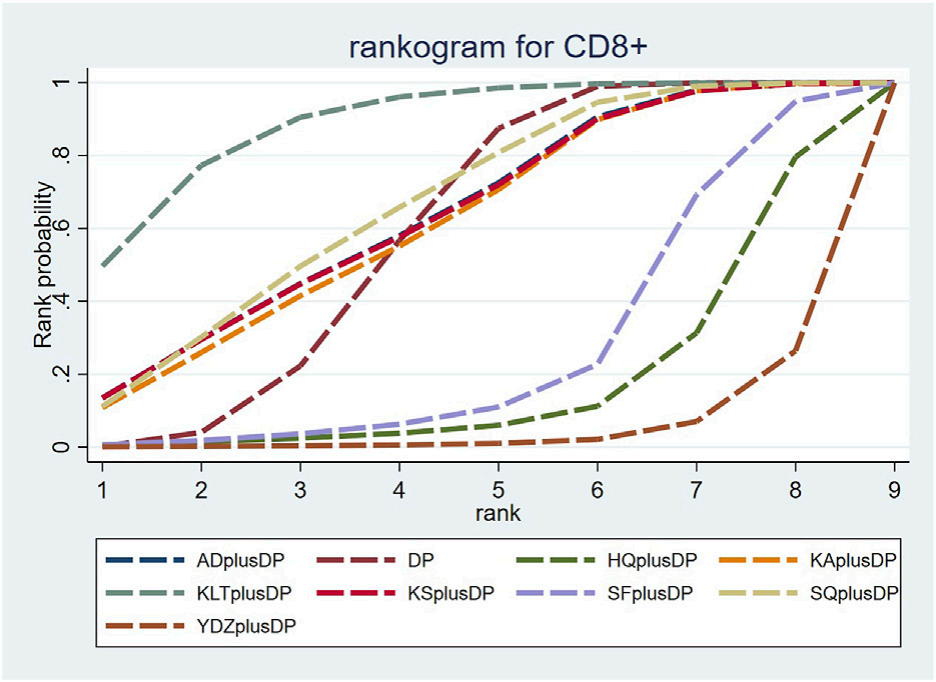
Cumulative probability line chart of CD8^+^.

Eighteen studies reported information on CD8^+^ levels. The results indicated that Kanglaite Injection plus DP (MD = 11.42, 95% CI: 1.45, 21.49) was more effective in improving CD8^+^ levels than Astragalus Injection plus DP. Shenfu Injection plus DP (MD = −8.59, 95% CI: −16.72, −0.60) was less effective in improving CD8^+^ levels compared to Kanglaite Injection plus DP. Brucea Javanica Oil Milk Injection plus DP was less effective in improving CD8^+^ levels compared to Aidi Injection plus DP (MD = −12.98, 95% CI: −24.37, −1.94), DP alone (MD = −12.60, 95% CI: −21.68, −3.49), Kangai Injection plus DP (MD = −12.77, 95% CI: −23.86, −1.70), Kanglaite Injection plus DP (MD = −15.93, 95% CI: −25.96, −6.00), Compound Kushen Injection plus DP (MD = −12.98, 95% CI: −24.29, −1.66), and Shenqi Fuzheng Injection plus DP (MD = −13.33, 95% CI: −23.85, −2.84). Other interventions did not show significant differences in pairwise comparisons (see Table 6). According to the cumulative probability results, Kanglaite Injection plus DP (SUCRAs: 88.96%), Shenqi Fuzheng Injection plus DP (SUCRAs: 66.40%), and Aidi Injection plus DP (SUCRAs: 63.35%) were likely to be the top three measures in improving CD8^+^ levels (see Figure 8).

**TABLE 6:** CD8^+^ league table.

ADplusDP	−0.39 (−7.13, 6.05)	−8.49 (−19.85, 2.67)	−0.23 (−9.49, 8.77)	2.94 (−4.86, 10.6)	−0.01 (−9.47, 9.25)	−5.66 (−15.45, 3.81)	0.33 (−8.22, 8.61)	−12.98 (−24.37, −1.94)
0.39 (−6.05, 7.13)	DP	−8.09 (−17.24, 1.09)	0.17 (−6.2, 6.48)	3.32 (−0.66, 7.42)	0.37 (−6.26, 7.03)	−5.27 (−12.31, 1.73)	0.73 (−4.5, 5.96)	**−12.6 (-21.68, -3.49)**
8.49 (−2.67, 19.85)	8.09 (−1.09, 17.24)	HQplusDP	8.25 (−2.85, 19.42)	**11.42 (1.45, 21.49)**	8.47 (−2.9, 19.77)	2.82 (−8.73, 14.32)	8.83 (−1.76, 19.37)	−4.52 (−17.46, 8.41)
0.23 (−8.77, 9.49)	−0.17 (−6.48, 6.2)	−8.25 (−19.42, 2.85)	KAplusDP	3.15 (−4.29, 10.78)	0.2 (−8.97, 9.4)	−5.46 (−14.88, 3.99)	0.55 (−7.64, 8.81)	**−12.77 (-23.86, -1.7)**
−2.94 (−10.6, 4.86)	−3.32 (−7.42, 0.66)	−11.42 (−21.49, −1.45)	−3.15 (−10.78, 4.29)	KLTplusDP	−2.94 (−10.78, 4.76)	**−8.59 (-16.72, -0.6)**	−2.6 (−9.28, 3.95)	**−15.93 (-25.96, -6)**
0.01 (−9.25, 9.47)	−0.37 (−7.03, 6.26)	−8.47 (−19.77, 2.9)	−0.2 (−9.4, 8.97)	2.94 (−4.76, 10.78)	KSplusDP	−5.64 (−15.33, 3.99)	0.35 (−8.12, 8.87)	**−12.98 (-24.29, -1.66)**
5.66 (−3.81, 15.45)	5.27 (−1.73, 12.31)	−2.82 (−14.32, 8.73)	5.46 (−3.99, 14.88)	8.59 (0.6, 16.72)	5.64 (−3.99, 15.33)	SFplusDP	6 (−2.7, 14.79)	−7.34 (−18.79, 4.19)
−0.33 (−8.61, 8.22)	−0.73 (−5.96, 4.5)	−8.83 (−19.37, 1.76)	−0.55 (−8.81, 7.64)	2.6 (−3.95, 9.28)	−0.35 (−8.87, 8.12)	−6 (−14.79, 2.7)	SQplusDP	**−13.33 (-23.85, -2.84)**
12.98 (1.94, 24.37)	12.6 (3.49, 21.68)	4.52 (−8.41, 17.46)	12.77 (1.7, 23.86)	15.93 (6, 25.96)	12.98 (1.66, 24.29)	7.34 (−4.19, 18.79)	13.33 (2.84, 23.85)	YDZplusDP

The bold values indicated that Kanglaite Injection plus DP (MD = 11.42, 95% CI: 1.45, 21.49) was more effective in improving CD8^+^ levels than Astragalus Injection plus DP. Shenfu Injection plus DP (MD = −8.59, 95% CI: −16.72, −0.60) was less effective in improving CD8^+^ levels compared to Kanglaite Injection plus DP. Brucea Javanica Oil Milk Injection plus DP was less effective in improving CD8^+^ levels.

#### 3.4.7 CD4^+^/CD8^+^

**FIGURE 9:**
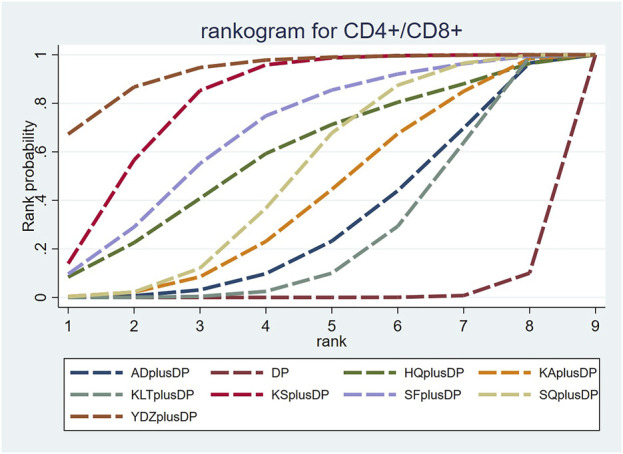
Cumulative probability line chart of CD4^+^/CD8^+^.

Twenty-one studies reported information on CD4^+^/CD8^+^ ratios. The results indicated that six regimens, including Kangai Injection plus DP (MD = 0.31, 95% CI: 0.03, 0.60), Kanglaite Injection plus DP (MD = 0.21, 95% CI: 0.04, 0.37), Compound Kushen Injection plus DP (MD = 0.63, 95% CI: 0.38, 0.87), Shenfu Injection plus DP (MD = 0.52, 95% CI: 0.12, 0.92), Shenqi Fuzheng Injection plus DP (MD = 0.37, 95% CI: 0.16, 0.58), and Brucea Javanica Oil Milk Injection plus DP (MD = 0.80, 95% CI: 0.41, 1.19), significantly outperformed DP alone in improving CD4^+^/CD8^+^ ratios, and the differences were statistically significant. Compound Kushen Injection plus DP (MD = 0.41, 95% CI: 0.12, 0.71) was more effective in improving CD4^+^/CD8^+^ ratios than Kanglaite Injection plus DP. In comparison to Aidi Injection plus DP (MD = 0.39, 95% CI: 0.02, 0.74), DP alone (MD = 0.80, 95% CI: 0.41, 1.19), Kangai Injection plus DP (MD = 0.49, 95% CI: 0.01, 0.97), and Kanglaite Injection plus DP (MD = 0.59, 95% CI: 0.18, 1.02), Brucea Javanica Oil Milk Injection plus DP was more effective in improving CD4^+^/CD8^+^ ratios. Other interventions did not show significant differences in pairwise comparisons (see Table 7). According to the cumulative probability results, Brucea Javanica Oil Milk Injection plus DP (SUCRAs: 93.13%), Compound Kushen Injection plus DP (SUCRAs: 81.25%), and Shenfu Injection plus DP (SUCRAs: 67.75%) were likely to be the top three measures in improving CD4^+^/CD8^+^ ratios (see Figure 9).

**TABLE 7:** CD4^+^/CD8^+^ league table.

ADplusDP	−0.24 (−0.51, 0.03)	0.21 (−0.35, 0.77)	0.07 (−0.32, 0.46)	−0.03 (−0.35, 0.28)	0.39 (0.02, 0.74)	0.28 (−0.19, 0.76)	0.13 (−0.21, 0.47)	0.56 (0.09, 1.03)
0.24 (−0.03, 0.51)	DP	0.45 (−0.05, 0.95)	**0.31 (0.03, 0.6)**	**0.21 (0.04, 0.37)**	**0.63 (0.38, 0.87)**	**0.52 (0.12, 0.92)**	**0.37 (0.16, 0.58)**	**0.8 (0.41, 1.19)**
−0.21 (−0.77, 0.35)	−0.45 (−0.95, 0.05)	HQplusDP	−0.14 (−0.71, 0.43)	−0.24 (−0.77, 0.28)	0.18 (−0.38, 0.73)	0.07 (−0.56, 0.71)	−0.08 (−0.62, 0.46)	0.35 (−0.28, 0.98)
−0.07 (−0.46, 0.32)	−0.31 (−0.6, −0.03)	0.14 (−0.43, 0.71)	KAplusDP	−0.1 (−0.44, 0.22)	0.32 (−0.07, 0.69)	0.21 (−0.28, 0.7)	0.06 (−0.29, 0.42)	**0.49 (0.01, 0.97)**
0.03 (−0.28, 0.35)	−0.21 (−0.37, −0.04)	0.24 (−0.28, 0.77)	0.1 (−0.22, 0.44)	KLTplusDP	**0.41 (0.12, 0.71)**	0.31 (−0.12, 0.75)	0.15 (−0.1, 0.44)	**0.59 (0.18, 1.02)**
−0.39 (−0.74, −0.02)	−0.63 (−0.87, −0.38)	−0.18 (−0.73, 0.38)	−0.32 (−0.69, 0.07)	−0.41 (−0.71, −0.12)	KSplusDP	−0.11 (−0.57, 0.37)	−0.26 (−0.58, 0.08)	0.17 (−0.28, 0.64)
−0.28 (−0.76, 0.19)	−0.52 (−0.92, −0.12)	−0.07 (−0.71, 0.56)	−0.21 (−0.7, 0.28)	−0.31 (−0.75, 0.12)	0.11 (−0.37, 0.57)	SFplusDP	−0.15 (−0.6, 0.3)	0.28 (−0.28, 0.83)
−0.13 (−0.47, 0.21)	−0.37 (−0.58, −0.16)	0.08 (−0.46, 0.62)	−0.06 (−0.42, 0.29)	−0.15 (−0.44, 0.1)	0.26 (−0.08, 0.58)	0.15 (−0.3, 0.6)	SQplusDP	0.43 (−0.01, 0.87)
−0.56 (−1.03, −0.09)	−0.8 (−1.19, −0.41)	−0.35 (−0.98, 0.28)	−0.49 (−0.97, −0.01)	−0.59 (−1.02, −0.18)	−0.17 (−0.64, 0.28)	−0.28 (−0.83, 0.28)	−0.43 (−0.87, 0.01)	YDZplusDP

The bold values indicated that six regimens, including Kangai Injection plus DP (MD = 0.31, 95% CI: 0.03, 0.60), Kanglaite Injection plus DP (MD = 0.21, 95% CI: 0.04, 0.37), Compound Kushen Injection plus DP (MD = 0.63, 95% CI: 0.38, 0.87), Shenfu Injection plus DP (MD = 0.52, 95% CI: 0.12, 0.92), Shenqi Fuzheng Injection plus DP (MD = 0.37, 95% CI: 0.16, 0.58), and Brucea Javanica Oil Milk Injection plus DP (MD = 0.80, 95% CI: 0.41, 1.19), significantly outperformed DP alone in improving CD4^+^/CD8^+^ ratios.

#### 3.4.8 Adverse reactions

Adverse reactions included leukopenia, hemoglobinopenia, thrombocytopenia, erythropenia, neutropenia, nausea and vomiting, diarrhea, constipation, abdominal pain, alopecia, abnormal liver function, abnormal renal function, phlebitis, cardiotoxicity, neurotoxicity, fatigue, oral ulcer, rash, mucositis, tinnitus, and hypotension. Sixty-four randomized controlled trials recorded the above adverse effects. Overall, Xiaoaiping Injection plus DP had the highest incidence of leukopenia (77.59%); Shenfu Injection plus DP had the highest incidence of hemoglobinopenia (48.31%), thrombocytopenia (50.00%), neutropenia (12.71%), nausea and vomiting (66.10%), constipation (44.07%), abnormal liver function (15.25%), and abnormal renal function (10.17%); Cinobufacini Injection plus DP had the highest incidence of diarrhea (9.72%); Kangai Injection plus DP had the highest incidence of alopecia (15.37%) and neurotoxicity (7.79%); Aidi Injection plus DP had the highest incidence of phlebitis (1.85%) and oral ulcers (3.09%); Shenqi Fuzheng Injection plus DP had the highest incidence of fatigue (6.43%). In addition, the incidence of erythropenia (0.41%), abdominal pain (13.89%), cardiotoxicity (0.22%), rash (0.87%), mucositis (0.65%), tinnitus (4.17%), and hypotension (0.87%) was reported in only one study each, respectively. The results are presented in Table 8 below.

**TABLE 8:** Incidence of adverse effects.

Interventions	ADplusDP	SQplusDP	KSplusDP	KAplusDP	YDZplusDP	SFplusDP	SMplusDP	KLTplusDP	HCSplusDP	XAPplusDP
**Sample size**	647	249	484	462	187	118	50	129	72	58
**Leukopenia**	40.80% (264/647)	37.35% (93/249)	33.88% (164/484)	26.41% (122/462)	1.07% (2/187)	57.63% (68/118)	48% (24/50)	24.03% (31/129)	30.56% (22/72)	77.59% (45/58)
**Hemoglobinopenia**	2.78% (18/647)	19.28% (48/249	5.99% (29/484)	8.23% (38/462)		48.31% (57/118)		3.10% (4/129)		39.66% (23/58)
**Thrombocytopenia**	9.12% (59/647)	18.86% (47/249)	12.40% (60/484)	5.84% (27/462)		50.00% (59/118)		3.10% (4/129)	1.39% (1/72)	39.66% (23/58)
**Erythropenia**			0.41% (2/484)							
**Neutropenia**						12.71% (15/118)		7.75% (10/129)		
**Nausea and vomiting**	49.46% (320/647)	30.12% (75/249)	42.98% (208/484)	38.31% (177/462)	38.50% (72/187)	66.10% (78/118)	60% (30/50)	51.94% (67/129)	18.06% (13/72)	51.72% (30/58)
**Diarrhea**	1.08% (7/647)	1.61% (4/249)		0.22% (1/462)		4.24% (5/118)			9.72% (7/72)	
**Constipation**		4.82% (12/249)	1.86% (9/484)			44.07% (52/118)				
**Abdominal pain**									13.89% (10/72)	
**Alopecia**	0.46% (3/647)	9.24% (23/249)		15.37% (71/462)						
**Abnormal liver function**	5.72% (37/647)	4.82% (12/249)	2.48% (12/484)	1.73% (8/462)		15.25% (18/118)		13.95% (18/129)		8.62% (5/58)
**Abnormal renal function**	0.62% (4/647)	3.21% (8/249)	0.41% (2/484)	1.08% (5/462)		10.17% (12/118)				
**Phlebitis**	1.85% (12/647)	0.40% (1/249)								
**Cardiotoxicity**				0.22% (1/462)						
**Neurotoxicity**	7.57% (49/647)	2.41% (6/249)	1.45% (7/484)	7.79% (36/462)						
**Fatigue**		6.43% (16/249)	0.83% (4/484)	2.16% (10/462)						
**Oral ulcer**	3.09% (20/647)	0.80% (2/249)				1.69% (2/118)				
**Rash**				0.87% (4/462)						
**Mucositis**				0.65% (3/462)						
**Tinnitus**									4.17% (3/72)	
**Hypotension**				0.87% (4/462)						

AD, aidi injection; SQ, shenqifuzheng injection; SF, shenfu injection; KS, compound kushen injection; KA, kangai injection; YDZ, brucea javanica oil milk injection; SM, shenmai injection; HCS, cinobufacini injection; XAP, xiaoaiping injection; KLT, kanglaite injection.

### 3.5 Cluster analysis

**FIGURE 10:**
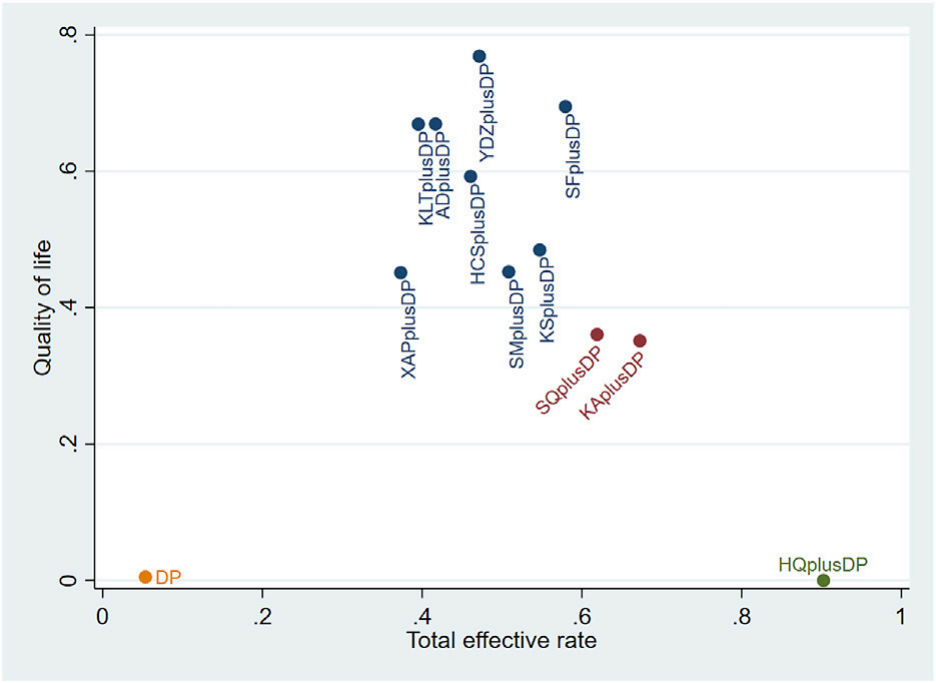
Response rate and QoL cluster plot.

**FIGURE 11:**
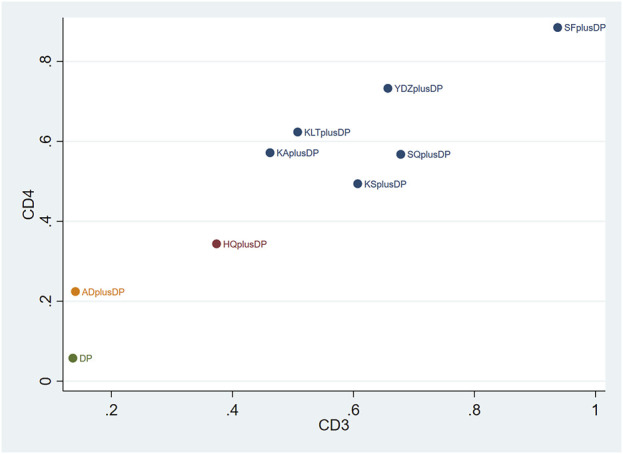
CD3^+^ and CD4^+^ cluster plot.

**FIGURE 12:**
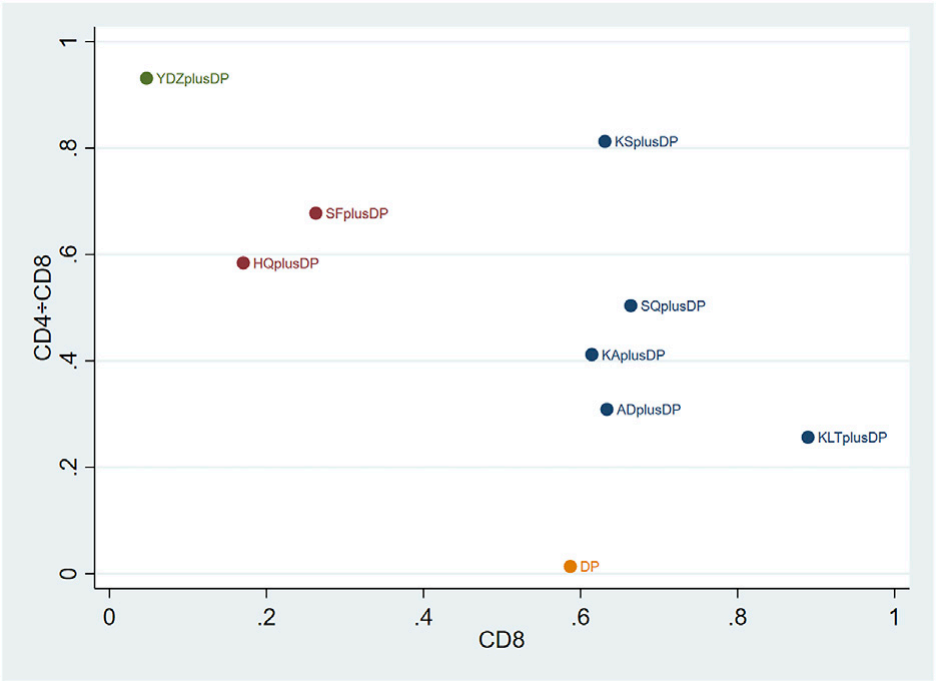
CD8^+^ and CD4^+^/CD8^+^ cluster plot.

The two-dimensional clustering results showed that Astragalus Injection plus DP was the most effective in improving the response rate; Brucea Javanica Oil Milk Injection plus DP was the most effective in improving QoL, while DP alone ranked the worst overall in improving the response rate and QoL (see Figure 10). Shenfu Injection plus DP was the most effective in improving CD3^+^ and CD4^+^ levels, while DP alone ranked the worst overall in increasing CD3^+^ and CD4^+^ levels (see Figure 11). Additionally, Kanglaite Injection plus DP was the most effective in improving CD8^+^ levels; Brucea Javanica Oil Milk Injection plus DP was the most effective in improving CD4^+^/CD8^+^ ratios, with DP alone ranking the worst overall in improving CD8^+^ levels and CD4^+^/CD8^+^ ratios (see Figure 12).

### 3.6 Publication bias

**FIGURE 13:**
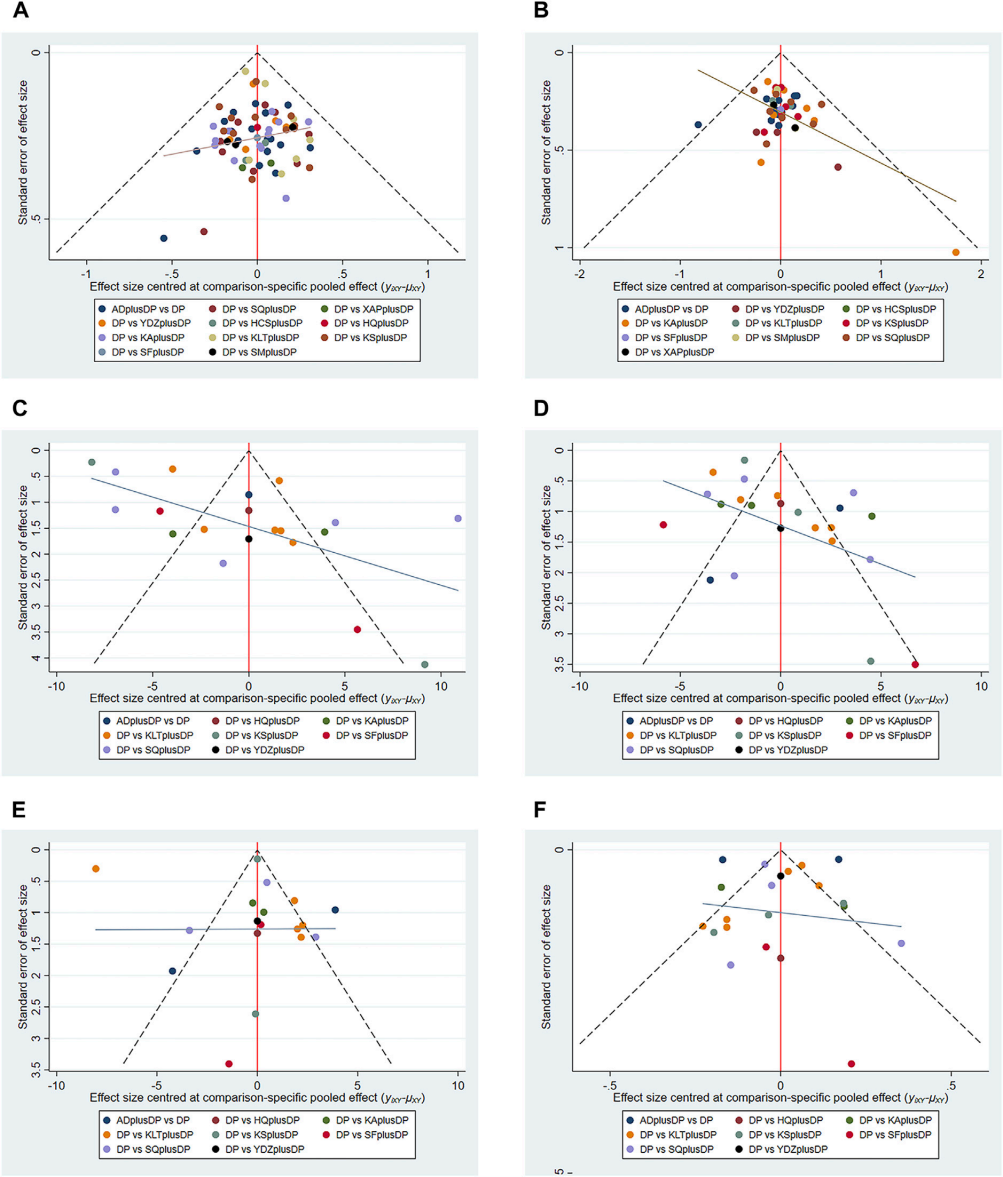
Funnel plot. **(A)** Response rate funnel plot; **(B)** QoL funnel plot; **(C)** CD3^+^ funnel plot; **(D)** CD4^+^ funnel plot; **(E)** CD8^+^ funnel plot; **(F)** CD4^+^/CD8^+^ funnel plot.

Funnel plots were used to assess the publication bias of all outcome indicators. The funnel plots for the response rate and CD8^+^ were symmetrical on both sides, indicating no publication bias. However, the funnel plots for QoL, CD3^+^, CD4^+^, and CD4^+^/CD8^+^ were asymmetrical, suggesting potential publication bias (see Figure 13).

## 4 Discussion

To the best of our knowledge, this is the first Network Meta-Analysis (NMA) comparing the efficacy and safety of different CHIs in combination with DP for NSCLC. This NMA analyzed the latest data from 85 eligible RCTs. Our results indicate that Astragalus Injection plus DP was the most effective intervention in improving the response rate; Brucea Javanica Oil Milk Injection plus DP was the most effective intervention in enhancing QoL; Shenfu Injection plus DP was the most effective intervention in improving CD3^+^ and CD4^+^ levels; Kanglaite Injection plus DP was the most effective intervention in increasing CD8^+^ levels, and Brucea Javanica Oil Milk Injection plus DP was the most effective intervention in improving CD4^+^/CD8^+^ ratios.

In terms of improving RR, Astragalus Injection has a clear advantage. Astragalus Injection is composed of astragalus extract, whose main efficacy components include polysaccharides, saponins and flavonoids (Hui et al., 2002). Modern pharmacological studies show that astragalus polysaccharides can block the liver cancer cell cycle by up-regulating LC3B protein expression and down-regulating LC3A and P62 protein expression in anti-liver cancer cell proliferation experiments, further inducing mitochondrial apoptosis and promoting cell autophagy and apoptosis. Astragalus polysaccharides can inhibit the increase of autophagosomes in xanthine oxidase-induced lung cancer cells during LC3B and P62 protein expression. Astragalus polysaccharides can not only enhance human cell autophagy but also inhibit cancer cell autophagy (Fang and Lijiang, 2020). In addition, AS-IV in astragaloside (AS) can increase the ratio of pro-apoptotic protein (Bax) to anti-apoptotic protein B-cell lymphoma-2 (Bcl-2), upregulate the expression of caspase-3 and caspase-9 in the caspase family, and induce endogenous apoptosis in various types of cancers, including colorectal cancer, breast cancer, lung cancer, hepatocellular carcinoma, *etc.* (Suying et al., 2016; Lijun et al., 2017; Liwei et al., 2019; SUN et al., 2019; Fang and Lijiang, 2020; Xiang et al., 2020), which is concentration- and time-dependent. Astragalin can increase reduced spleen and thymus indexes caused by Lewis cells and regulate XBP1-mediated endoplasmic reticulum stress response to regulate immunity and inhibit tumor growth (Bing et al., 2019). The latest review shows that there are currently no pharmacokinetic studies on Astragalus Injection (Yixiang et al., 2023). Although it is found that Astragalus Injection is beyond compare in improving the response rate, there is only one article on it. Therefore, the ranking of its results shall be interpreted with caution. It is expected that there will be more RCTs on Astragalus Injection in the future.

Regarding the improvement of QoL and CD4^+^/CD8^+^ ratios, Brucea Javanica Oil Milk Injection demonstrated significant effectiveness. The primary medicinal component of Brucea Javanica Oil Milk Injection is Brucea Javanica Oil Milk (Liancui and Xuedong, 2013). A relevant pharmacological study has shown that Brucea Javanica oil emulsion acts as a non-specific anticancer drug affecting various phases of the cell cycle, leading to the killing and inhibition of tumor cells in G0, G1, S, G2, and M phases. It significantly inhibits the synthesis of DNA, RNA, and protein in tumor cells and interferes with the formation of peptide bonds (Peigeng, 2002). Additionally, Zhu Xiangliang et al. confirmed that Brucea Javanica oil inhibited the proliferation and migration of lung cancer A549 cells in a dose-dependent manner, induced the aggregation of green fluorescence of autophagy-related protein (LC)3, and promoted the transformation from LC 3-I to LC 3-II (Xiangliang and Pinhua, 2018). A human pharmacokinetic study showed that the estimated terminal elimination half-life (t_1/2_) of the concentration of oleic acid in human plasma (the index component in brucea javanica oil milk) was 12.14 ± 6.42 h, time to peak (T_max_) was 1.08 ± 0.19 h, peak concentration (C_max_) was 95.20 ± 29.10 mg L^-1^, and area under the curve (AUC_0-12_) was 370.89 ± 70.71 mg h L^-1^. The recommended clinical dose is 100 mL once daily (Yan, 2009).

For the enhancement of CD3^+^ and CD4^+^ levels, Shenfu Injection demonstrates significant advantages. Shenfu Injection is derived from the extract of red ginseng and aconite (black aconite tablet), with its main components being ginsenoside in red ginseng and aconite alkaloids in aconite (Mingyu et al., 2018). A relevant pharmacological study indicates that ginsenoside Rh1 in Shenfu Injection can significantly increase the spleen and thymus indices, enhance macrophage phagocytosis, and promote the proliferation of T lymphocytes in mice. Both ginsenoside Rg1 and Rh1 can stimulate the release of NO and improve macrophage phagocytosis (Yi et al., 2002; Caijun et al., 2009). Aconitum alkaloids in Shenfu Injection can directly inhibit or destroy lung cancer cells (Lew, 2019; Sehouli et al., 2019). Liu Qiang et al. also found that Shenfu Injection can enhance the immune function of patients by increasing the Treg ratio (Qiang et al., 2018). A pharmacokinetic study in rats showed that t_1/2_ was 8.685 min, terminal elimination rate constant (Ke) was 0.08 min^-1^, CL was 1.417 L min^-1^ kg^-1^, and AUC_0-t_ was 12.63 mg L^-1^ min^-1^. The study results showed that the apparent pharmacokinetic process of the plasma concentration of Shenfu Injection injected intravenously into rats was fitted to a one-compartment model (Ting et al., 2012).

Kanglaite Injection clearly demonstrates its benefits in improving CD8^+^ levels. Kanglaite Injection is primarily composed of coix lachryma-jobi oil extracted from coix lachryma-jobi (Zhiyong and Xuhe, 2009). A related pharmacological study suggests that Kanglaite Injection can prevent tumor cells from entering the G0 and G phases by targeting the G2+M phase and inducing tumor cell apoptosis (Yin and Tingzhang, 2002). Zhang Aiqin et al. investigated the impact of Kanglaite Injection on anti-tumor and immune functions by measuring the activities of TNF-α, IL-1, and IL-6 in the peripheral blood of mice. The results indicated that Kanglaite Injection had a protective effect on the immune organs and immune function of the body while also eliminating tumor cells (Aiqin et al., 2007). A pharmacokinetic study in rats showed that in Kanglaite Injection 10 and 5 mL/kg groups, t_1/2α_(h) was 0.481 ± 0.168 and 0.322 ± 0.109 respectively; t_1/2β_(h) was 1.452 ± 0.776 and 1.384 ± 0.404 respectively; C_max_ (mmol/L) was 8.532 ± 1.031 and 5.418 ± 0.764 respectively; AUC_0-t_ (mmol/L•h) was 13.248 ± 3.692 and 5.339 ± 1.219 respectively; apparent volume of distribution (V_d_) was (1.030 ± 0.131) and (0.756 ± 0.150) L^2^/(kg·mol) respectively; and CL was (0.838 ± 0.319) and (0.975 ± 0.330)L^2^/(kg·mol·h) respectively, wherein, t_1/2α_ was 0.135 h and t_1/2β_ was 15.84 h. The study results showed that the pharmacokinetic process of Kanglaite Injection injected intravenously in rats was fitted to a two-compartment open model (Fei et al., 2009).

### 4.1 Limitations

There are several unavoidable limitations to the current NMA. First, the relatively small number of partial intervention studies included in this NMA may have had some impact on the conclusions. Second, despite the inclusion of randomized controlled studies, some articles lacked blinding, which could have introduced bias. Third, due to limitations in the extracted data from the included studies, it was not possible to perform more detailed subgroup analyses, which may have influenced the final results. Therefore, it is recommended that RCTs be registered in advance to ensure transparency in the timeline and improve the methodological quality. Additionally, RCTs should be conducted in accordance with the latest clinical diagnostic and therapeutic guidelines. Furthermore, RCTs involving cancer patients should focus on long-term and clinically meaningful endpoints. Given the aforementioned limitations, more rigorous, high-quality RCTs are needed to confirm the efficacy of CHIs in combination with DP for the treatment of NSCLC patients.

## 5 Conclusion

In summary, the current evidence suggests that CHIs in combination with DP may offer more benefits to NSCLC patients compared to DP alone. Among the 11 interventions, Astragalus Injection plus DP, Brucea Javanica Oil Milk Injection plus DP, Shenfu Injection plus DP, and Kanglaite Injection plus DP appear to be the preferred treatment options for NSCLC. However, due to limitations in the number and quality of articles, further research is needed in the form of high-quality, large-scale, double-blind RCTs to confirm the findings of this NMA.
